# Endogenous Ceramide 24:1 Constrains Th17‐Driven Neutrophilic Inflammation by Antagonizing EP2 Signaling

**DOI:** 10.1002/advs.202520695

**Published:** 2026-03-13

**Authors:** Huan Liu, Abudureyimujiang Aili, Zheng Kuang, Liting Cao, Zemin Li, Ying Shang, Yingying Ge, Tingting Hu, Yongchang Sun, Wuli Zhao, Rong Jin, Chun Chang

**Affiliations:** ^1^ Department of Respiratory and Critical Care Medicine Peking University Third Hospital Beijing China; ^2^ Department of Medical Oncology and Radiation Sickness Peking University Third Hospital Beijing China; ^3^ Department of Immunology Peking University Health Science Center Peking University Center for Human Disease Genomics Beijing China; ^4^ State Key Laboratory of Respiratory Health and Multimorbidity Key Laboratory of Antibiotic Bioengineering Laboratory of Oncology Institute of Medicinal Biotechnology Ministry of Health Chinese Academy of Medical Sciences & Peking Union Medical College Beijing China; ^5^ Department of Immunology School of Basic Medical Sciences Medicine Innovation Center for Fundamental Research on Major Immunology‐Related Diseases NHC Key Laboratory of Medical Immunology (Peking University) Peking University Beijing China

**Keywords:** ceramide, neutrophilic asthma, sphingolipids, Th17 cells

## Abstract

Dysregulated chronic inflammation underlies a spectrum of severe asthma phenotypes, among which neutrophilic asthma (NA) represents a treatment‐recalcitrant endotype characterized by Th17‐driven airway inflammation and steroid resistance. Although lipid mediators are known to play dual roles in promoting and resolving inflammation, the lipid species governing the Th17‐neutrophil axis in NA remain unknown. Here, through integrated lipidomic profiling of clinical samples (exhaled breath condensate, plasma, sputum) from an NA cohort and a murine model of Th17‐driven airway inflammation, a deficiency in very‐long‐chain ceramides, notably Cer24:1, was identified. This reduction correlated with disease severity and neutrophilic inflammation. In vivo, Cer24:1 supplementation alleviated airway hyperresponsiveness and neutrophilic infiltration, while *Smpd1* knockout mice—with impaired ceramide generation—displayed exacerbated Th17 pathology. Using structure‐guided molecular docking, surface plasmon resonance, and functional assays, Cer24:1 was shown to directly target the prostaglandin E2 receptor EP2 on CD4^+^ T cells. This interaction suppressed JAK2–STAT3 signaling and RORγt‐driven Th17 differentiation. Notably, PGE_2_ competitively reversed Cer24:1's protective effects, further supporting EP2‐dependent modulation. Our results reveal Cer24:1 as an endogenous pro‐resolving lipid that constrains neutrophilic inflammation via direct modulation of the EP2–STAT3 axis in Th17 cells, providing a new metabolic checkpoint and potential therapeutic strategy for severe neutrophilic asthma.

## Graphical abstract

Cer24:1 levels are reduced in neutrophilic asthma and inversely correlate with disease severity and airway neutrophilia. Restoring Cer24:1 suppresses pathogenic Th17 differentiation by engaging EP2 on CD4^+^ T cells, thereby dampening the JAK2–STAT3–RORγt axis and reducing IL‐17 production. Cer24:1 supplementation alleviates neutrophil recruitment and airway inflammation, revealing a ceramide‐based metabolic checkpoint for steroid‐refractory neutrophilic asthma.

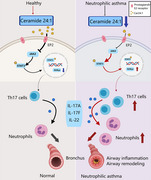

## 1 Introduction

Inflammation constitutes a fundamental and highly orchestrated immune response essential for host defense against pathogens and for tissue repair [1]. However, dysregulated or chronic inflammation is a cornerstone of the pathogenesis of a myriad of diseases, including asthma [2, 3]. Type 2 cytokines (such as IL‐4, IL‐5, and IL‐13) promote hallmark features of asthma such as eosinophilia, mucus hypersecretion, airway hyperresponsiveness (AHR), IgE production, and susceptibility to exacerbations. However, only ∼50% of patients with asthma have this type 2 inflammation signature. Neutrophilic asthma (NA) represents the most severe and glucocorticoid‐resistant non‐type 2 asthma endotype, accounting for ∼20%–30% of poorly controlled cases. CD4^+^ T helper 17 (Th17) cells play a pivotal role in orchestrating neutrophilic inflammation through the production of key cytokines (such as IL‐17A, IL‐17F, G‐CSF), leading to sustained neutrophil recruitment and activation [4, 5]. Once infiltrated, neutrophils release proteases, reactive oxygen species, and additional proinflammatory mediators that further amplify tissue damage and cytokine production, thereby establishing a self‐propagating cycle of inflammation that underlies the chronicity and steroid resistance often observed in neutrophilic asthma [4, 6, 7]. Critically, the persistence of this inflammatory circuit suggests the involvement of deeper regulatory mechanisms.

In addition to protein‐based cytokines and chemokines, lipid mediators (LMs) have emerged as pivotal orchestrators of the inflammatory cascade, functioning not only as potent proinflammatory signals (e.g., prostaglandins and leukotrienes) but also as specialized pro‐resolving mediators (SPMs, e.g., resolvins, protectins, and maresins) that actively terminate inflammation and promote tissue homeostasis [8, 9, 10, 11]. Emerging evidence underscores the critical role of lipid inflammatory mediators in shaping CD4^+^ T‐cell polarization, with profound influences on the development and function of Th17 cells [11, 12]. Dysregulation of sphingolipids has been implicated in asthma [13, 14]. In neutrophilic asthma, a Th17‐driven circuit of neutrophil recruitment and activation is well‐established; however, the deeper regulatory mechanisms, particularly how specific sphingolipids fine‐tune this pathogenic loop, remain poorly defined.

Ceramides are the core building blocks of sphingolipids and are composed of a sphingoid base amide‐linked to a fatty acyl chain [15, 16]. Importantly, the biological effects of ceramides are strongly influenced by acyl‐chain length [17]: long‐chain ceramides (LC‐Cers; typically C16–C20) have frequently been linked to proinflammatory signaling and stress responses [18], whereas very‐long‐chain ceramides (VLC‐Cers; typically ≥ C22) more often support membrane organization and barrier integrity [19]. Thus, changes in the balance between LC‐Cers and VLC‐Cers may reshape immune cell activation and inflammatory outcomes [15, 17]. However, whether endogenous ceramide species with distinct acyl chain lengths actively function as anti‐inflammatory/pro‐resolving mediators in NA patients, and which specific species are involved, remains unclear.

To address this knowledge gap, we performed systematic lipidomic profiling in neutrophilic asthma and conducted a targeted functional screen of chain‐length‐specific sphingolipids. We identified Cer24:1 as reduced in NA and as selectively constraining Th17 differentiation while alleviating neutrophil‐dominant airway inflammation. Mechanistically, Cer24:1 antagonizes the EP2–JAK2–STAT3 axis, thereby attenuating EP2‐driven STAT3 activation and restraining the Th17–neutrophil program. Collectively, these findings suggest that Cer24:1 is a candidate pro‐resolving sphingolipid, extends arachidonic‐centered frameworks, and highlights a tractable lipid–immune axis for the treatment of steroid‐resistant NA.

## 2 Results

### 2.1 Cer24:1 Deficiency is Associated with Elevated Th17 Cells and Disease Severity in NA

**FIGURE 1:**
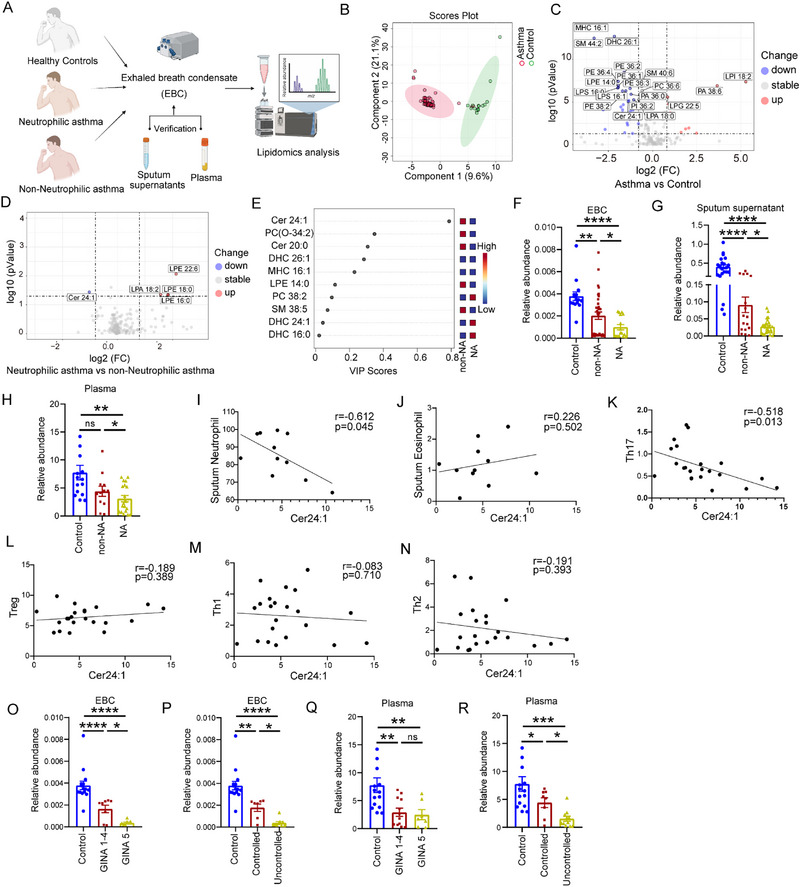
Targeted lipidomic analysis revealed a reduction in the abundance of the very‐long‐chain ceramide Cer24:1 in neutrophilic asthma patients. Targeted lipidomic analysis was conducted on exhaled breath condensate (EBC), plasma, and induced sputum samples from our cohort consisting of asthma patients and healthy controls. (A) Workflow of lipidomic analysis based on clinical specimens (created with BioRender.com under an academic license). (B) OPLS‐DA of phospholipids in the EBC of asthma patients (*n* = 60) and healthy controls (*n* = 16). (C) Volcano plots depicting the differential abundance of glycerophospholipids and sphingolipids in EBC samples between asthma patients (*n* = 60) and healthy controls (*n* = 16). For the volcano plots (C,D), *p* values were determined from Mann–Whitney U tests, and lipids with *p* < 0.05 and fold change ≥ 1.4 or ≤ 0.714 were considered as significantly altered. (D) Volcano plots depicting the differential abundance of glycerophospholipids and sphingolipids in EBC samples between neutrophilic asthma (NA) patients (*n* = 16) and non‐neutrophilic asthma (non‐NA) patients (*n* = 35). (E) VIP score plot of phospholipids between NA (*n* = 16) and non‐NA patients (*n* = 35). (F–H) Relative abundance of the very‐long‐chain ceramide Cer24:1 in EBC (HC, *n* = 16; non‐NA, *n* = 35; NA, *n* = 16), induced sputum supernatants (HC, *n* = 22; non‐NA, *n* = 17; NA, *n* = 24) and plasma (HC, *n* = 15; non‐NA, *n* = 12; NA, *n* = 19) from healthy controls, non‐NA patients and NA patients. (I–N) Correlations of plasma Cer24:1 levels with the proportions of neutrophils and eosinophils in the sputum and Th17, Th1, Th2, and Treg cells in the blood. (O,P) EBC Cer24:1 level in NA patients with GINA stages 1–4 (*n* = 8) or GINA stage 5 (*n* = 8) and with well‐controlled (ACT > 20, *n* = 7) or uncontrolled (ACT < 20, *n* = 9) symptoms. (Q,R) Plasma Cer24:1 level in NA patients at GINA stages 1–4 (*n* = 12) or GINA stage 5 (*n* = 7) with well‐controlled (ACT > 20, *n* = 8) or uncontrolled (ACT < 20, *n* = 11) symptoms. The data are presented as the mean ± standard error of the mean (SEM). Statistical analysis was performed via the Kruskal–Wallis test, and Spearman correlation analysis was conducted to evaluate associations. ^*^ *p <* 0.05, ^**^ *p <* 0.01, ^***^ *P <* 0.001, ^****^ *P <* 0.0001; ns, not significant.

To characterize the sphingolipid alterations in NA patients, we enrolled 173 patients with asthma and 53 healthy controls (Table S1). Exhaled breath condensate (EBC) samples were obtained from 60 patients with asthma and 16 healthy controls (Figure 1A). EBC is a noninvasive method that enables specific detection of airway metabolites while minimizing systemic and oral contamination. Targeted lipidomics revealed significant alterations in EBC lipid composition, with notable decreases in the levels of multiple ceramides, especially ceramide d18:1/24:1 (Cer24:1) (Figure 1B,C). Compared with non‐NA patients, NA patients also presented reduced levels of Cer24:1 and elevated levels of lysophosphatidic acid (LPA 18:2) and several lysophosphatidylethanolamines (LPEs) (Figure 1D), with Cer24:1 ranking among the top discriminators on the basis of the VIP score (Figure 1E).

To confirm these findings, the plasma and sputum samples of a larger cohort (defined here as participants with additional plasma and/or induced sputum available beyond the initial EBC cohort) were analyzed, and the results consistently revealed reduced Cer24:1 levels in NA patients (Figure S1A–C,F–H). The plasma Cer24:1 concentration of NA patients was inversely correlated with the proportions of both circulating Th17 cells and sputum neutrophils (Figure 1I,K) but was not correlated with the proportions of circulating Th1, Th2, Treg cells or sputum eosinophils. Similar inverse associations were also observed for other VLC‐Cers (such as Cer22:0 and Cer26:1), which were negatively correlated with Th17 cell (Figure S1D–K) and sputum neutrophil (Figure S1L–S) proportions. However, Cer24:1 exhibited the strongest inverse correlations with Th17 cells and neutrophils. In contrast, LC‐Cers did not exhibit significant correlations. Cer24:1 also demonstrated a robust ability to discriminate between NA patients and healthy controls (AUC > 0.8; Figure S1T–V).

Lower levels of Cer24:1 in EBC and plasma were also associated with increased asthma severity. Because the number of patients in individual GINA steps (1–4) was limited, we combined GINA steps 1–4 patients into one group and compared them with GINA step 5 patients to maintain adequate statistical power for subgroup analyses. NA patients with GINA step 5 treatment or with uncontrolled symptoms (asthma control test, ACT < 20) had significantly lower EBC Cer24:1 levels than those with milder disease or better disease control (Figure 1O,P). Similarly, the plasma Cer24:1 level was specifically decreased in NA patients with uncontrolled (ACT < 20) symptoms compared with those with well‐controlled (ACT > 20) symptoms (Figure 1Q,R). No significant differences in the ceramide levels in the sputum supernatant were detected across patient subgroups (Figure S1W,X). Notably, patients with low plasma Cer24:1 levels (below the median value of 3.87) were more likely to have experienced exacerbations in the past year (Figure S1Y).

### 2.2 Similar Alterations in the Proportion of Th17 Cells and Levels of Cer24:1 are Observed in HDM/LPS‐Induced NA Model Mice

**FIGURE 2:**
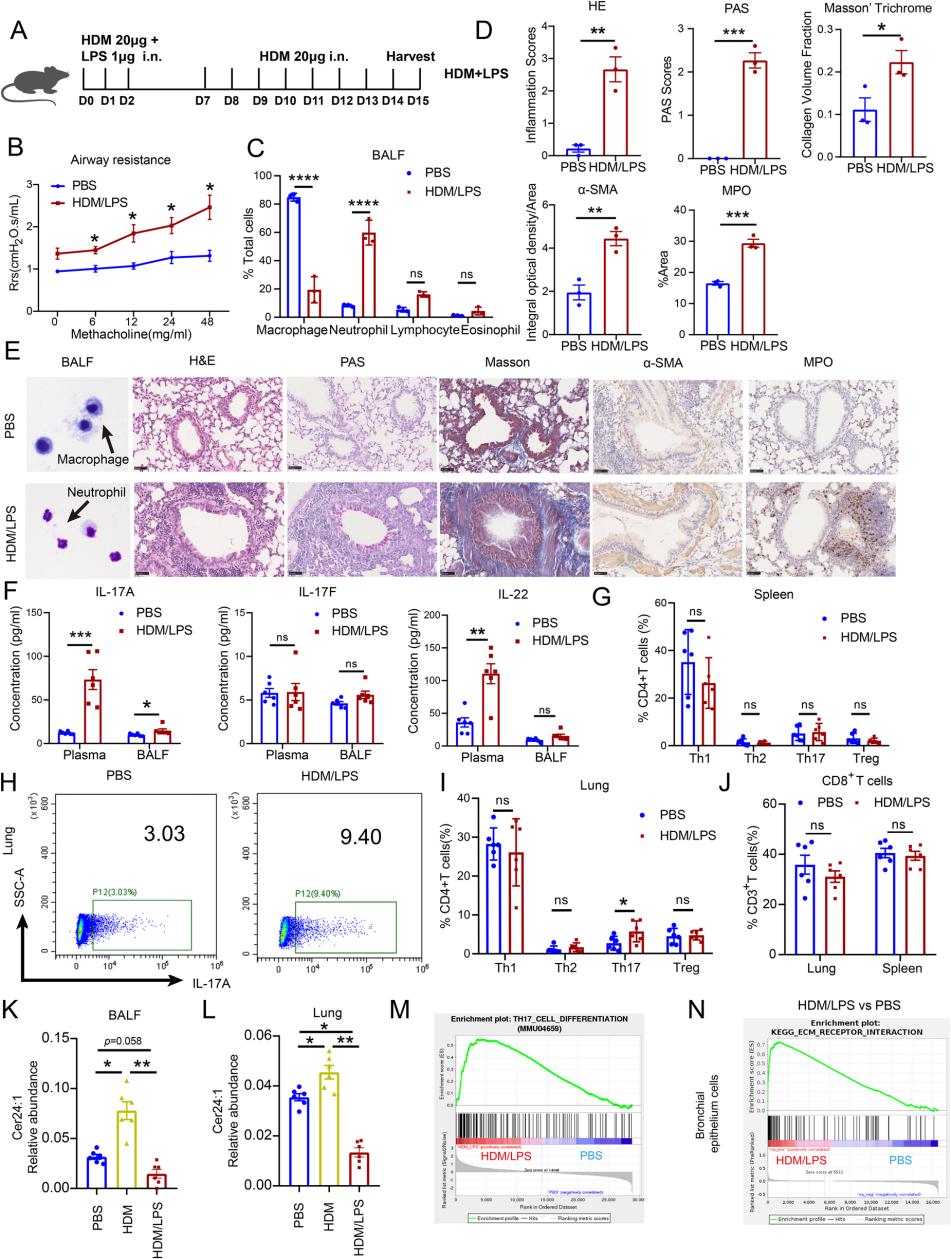
HDM combined with LPS induced Th17‐dominated neutrophilic asthma inflammation and decreased the level of Cer24:1 in mice. (A) Illustration of the experimental design. (B) Airway resistance in the different groups of mice in response to methacholine. Rrs represents the resistance of the respiratory system. (C) Percentages of neutrophils, eosinophils, macrophages and lymphocytes in the BALF (*n* = 3 mice/group). (D,E) Representative Wright–Giemsa staining of BALF, H&E, PAS, and Masson's trichrome staining and immunohistochemical staining of α‐SMA and MPO in lung sections, and a histogram showing the pathological scores. The scale bar is 50 µm. (*n* = 3 mice/group). (F) IL‐17A, IL‐17F, and IL‐22 concentrations in plasma and BALF were detected by CBA (*n* = 6 mice/group). (G–J) Flow cytometric analysis and statistical evaluation of Th1, Th2, Th17, Treg cells, and CD8^+^ T cells in the spleen and lung (*n* = 6 mice/group). The proportions of all CD4^+^ T‐cell subsets were analyzed by gating for Zombie Aqua^−^CD3^+^CD8^−^CD4^+^ T cells. The proportions of CD8^+^ T cells were analyzed by gating for Zombie Aqua^−^CD3^+^CD4^−^CD8^+^ T cells. (K,L) Relative abundance of Cer24:1 in BALF and lung tissue (*n* = 6 mice/group) from PBS‐treated mice (blue), HDM/LPS‐induced model mice (red), and HDM‐induced model mice (yellow). (M) Overview of the RNA‐seq and GSEA results identifying pathways affected by HDM/LPS stimulation, with the Th17 differentiation pathway being activated in HDM/LPS‐induced model mice (*n* = 6 mice/group). (N) GSEA of single‐cell sequencing data obtained from bronchial epithelial cells derived from the lungs of PBS‐treated and HDM/LPS‐induced model mice (*n* = 3 mice/group). The data are presented as the mean ± SEM. For two‐group comparisons (B–D, F,G,I), statistical analysis was performed using two‐tailed Student's *t* tests (or Mann–Whitney U tests for nonnormally distributed data). For three‐group comparisons (J,K), one‐way ANOVA followed by Tukey's post hoc test (or Kruskal–Wallis test with Dunn's multiple‐comparisons test for nonnormally distributed data) was used. ^*^ *p <* 0.05, ^**^ *p <* 0.01, ^***^ *p <* 0.001, ^****^ *P <* 0.0001; ns, not significant.

To simulate the pathological changes observed in NA patients, we established a mouse model of NA induced by HDM and lipopolysaccharide (LPS) (Figure 2A) [20, 21, 22]. The model mice presented increases in airway resistance (Figure 2B) and the proportion of neutrophils in the bronchoalveolar lavage fluid (BALF) (Figure 2C). Compared with those of control mice, histological analysis of lung sections revealed increased airway inflammation scores, periodic acid–Schiff (PAS) staining scores, and collagen deposition, as measured by Masson's trichrome staining (Figure 2D,E). Immunohistochemical staining for myeloperoxidase (MPO) and α‐smooth muscle actin (α‐SMA) in the HDM/LPS group further confirmed the increased neutrophil infiltration and airway remodeling (Figure 2D,E). The levels of Th17‐associated cytokines (IL‐17 and IL‐22) were elevated in both the plasma and BALF (Figure 2F). Flow cytometric analysis revealed an increased proportion of Th17 cells within the lung tissue but not in the spleen (Figure 2G–I); additionally, compared with that in the controls, the frequency of CD8^+^ T cells did not significantly change (Figure 2J). Lipidomic analysis of BALF and lung tissues from HDM/LPS‐induced mice revealed significant reductions in Cer24:1 and other VLC‐Cers, which were consistent with patient findings (Figure S2A,B). Compared with the HDM‐induced eosinophilic asthma model mice, the NA model mice presented a greater reduction in Cer24:1 levels in the lung tissue and BALF (Figure 2K,L), further highlighting the relevance of Cer24:1 in NA pathogenesis. Transcriptomic analysis revealed enrichment of Th17 differentiation pathways (Figure 2M), and single‐cell RNA sequencing of bronchial epithelial cells revealed the activation of airway remodeling pathways (Figure 2N).

### 2.3 Cer24:1 Supplementation Alleviates Neutrophilic Asthma

**FIGURE 3:**
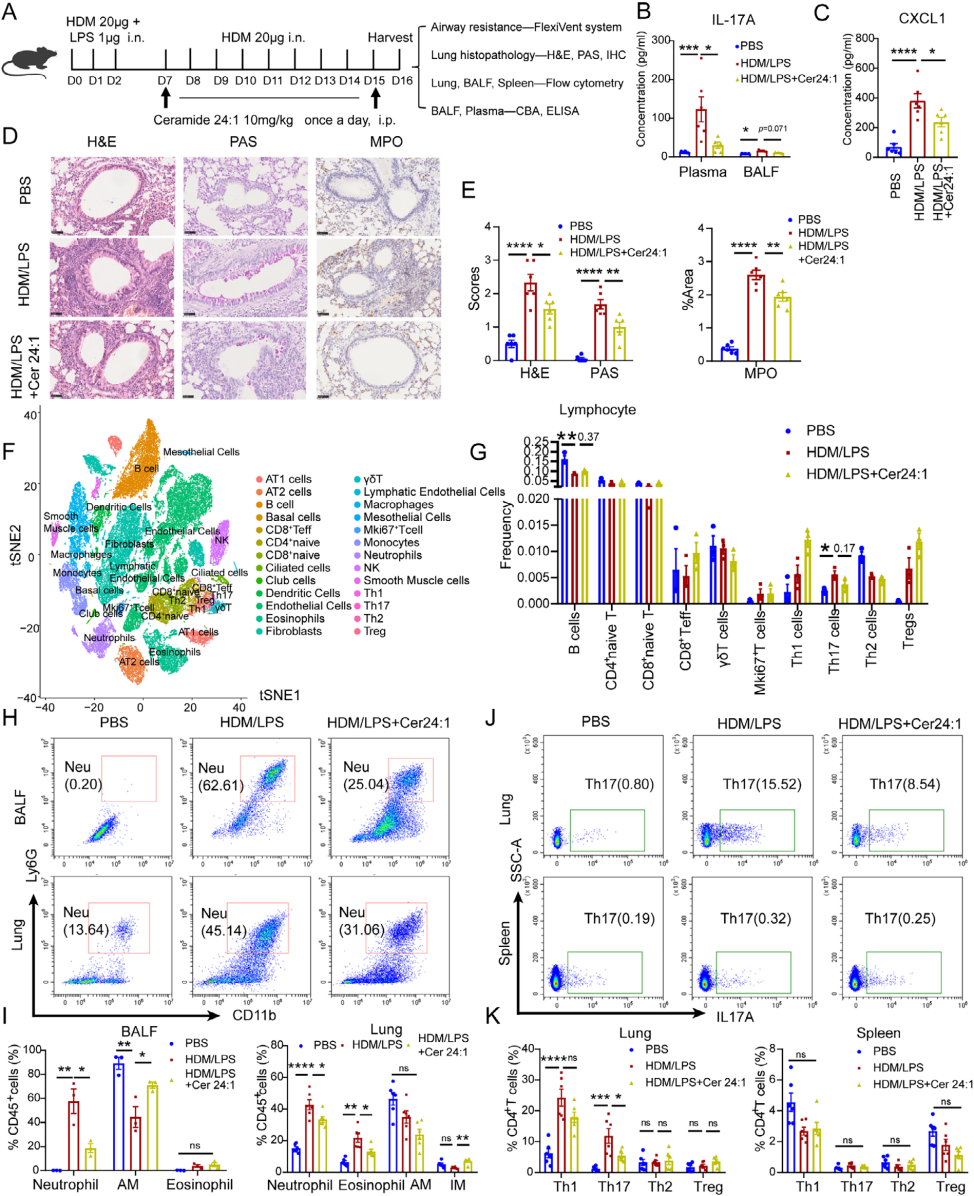
Cer24:1 treatment alleviated neutrophilic asthma in an HDM/LPS‐induced mouse model. (A) Illustration of the experimental design. (B,C) Concentrations of IL‐17A in the plasma and BALF were detected by a cytometric bead array (CBA), and CXCL1 in plasma was detected by ELISA. (D,E) Representative hematoxylin and eosin (H&E), PAS, and immunohistochemical staining of MPO in lung sections, along with a histogram of the pathological score. The scale bar indicates 50 µm. (F) T‐SNE plot of single‐cell RNA sequencing data from lung tissue. (G) Statistical analysis of the proportions of lymphocytes in the lung tissue of the three groups of mice via single‐cell RNA sequencing. (H,I) Flow cytometric analysis and statistical evaluation of neutrophils, eosinophils, alveolar macrophages (AM), and interstitial macrophages (IM) in the BALF (*n* = 3 mice/group) and lungs (*n* = 6 mice/group). The proportions of all myeloid cells were analyzed by gating on Zombie Aqua^−^CD45^+^ cells. (J–K) Flow cytometric analysis and statistical evaluation of Th1, Th2, Th17, and Treg cells in the lungs and spleen (*n* = 6 mice/group). The proportions of T‐cell subgroups were analyzed by gating on Zombie Aqua^−^CD3^+^CD8^−^CD4^+^ T cells. The data are presented as the mean ± SEM. For comparisons among three groups, statistical analysis was performed using one‐way ANOVA followed by Tukey's post hoc test (or Kruskal–Wallis test with Dunn's multiple comparisons test for nonnormally distributed data). ^*^ *p <* 0.05, ^**^ *p <* 0.01, ^***^ *p <* 0.001, ^****^ *p <* 0.0001; ns, not significant.

To evaluate the therapeutic potential of Cer24:1, HDM/LPS‐induced NA model mice were intraperitoneally injected with Cer24:1 (10 mg/kg) or DMSO (Figure 3A). Lipidomic analysis confirmed elevated Cer24:1 levels in the treated mice (Figure S3A). Although airway resistance remained unchanged (Figure S3B), Cer24:1 treatment significantly reduced the plasma levels of IL‐17A and the neutrophil chemoattractant CXCL1 (Figure 3B,C), whereas the levels of IL‐22 and IL‐17F remained unaffected (Figure S3C,D). Histopathological assessments revealed reduced airway inflammation, mucus secretion, and MPO‐positive neutrophil infiltration in Cer24:1‐treated mice (Figure 3D,E). Single‐cell RNA sequencing revealed a reduced proportion of Th17 cells in the lungs of Cer24:1‐treated mice (Figure 3F,G; Figure S3E–G), and flow cytometry confirmed fewer neutrophils in both the BALF and the lungs (Figure 3H,I) and fewer Th17 cells but no changes in the Th1, Th2, or Treg populations in the lungs (Figure 3J–K). Interestingly, the number of eosinophils in the lungs was also modestly reduced (Figure 3H,I), suggesting that Cer24:1 may partially affect eosinophil recruitment through non‐Th2‐mediated mechanisms.

Single‐cell RNA sequencing of the airway epithelium revealed suppression of extracellular matrix (ECM)‐related pathways in Cer24:1‐treated mice (Figure S3H), which was consistent with reduced collagen and fibronectin levels (Figure S3I–K). Notably, α‐SMA expression remained unchanged, suggesting the selective targeting of ECM turnover rather than myofibroblast activation. Cer24:1 treatment also decreased the MMP‐9 and TIMP‐1 concentrations in the BALF and plasma (Figure S3L,M), indicating systemic suppression of matrix remodeling.

In contrast, Cer24:1 had no therapeutic effect on HDM‐induced eosinophilic asthma. AHR, inflammation, mucus secretion, eosinophil infiltration, and Th2 responses remained unchanged (Figure S4A–J). These results indicate that Cer24:1 specifically targets Th17‐driven neutrophilic inflammation and not eosinophilic asthma.

### 2.4 *Smpd1* Deficiency Accelerated Th17‐Dominated Inflammation in HDM/LPS‐Induced NA Model Mice

**FIGURE 4:**
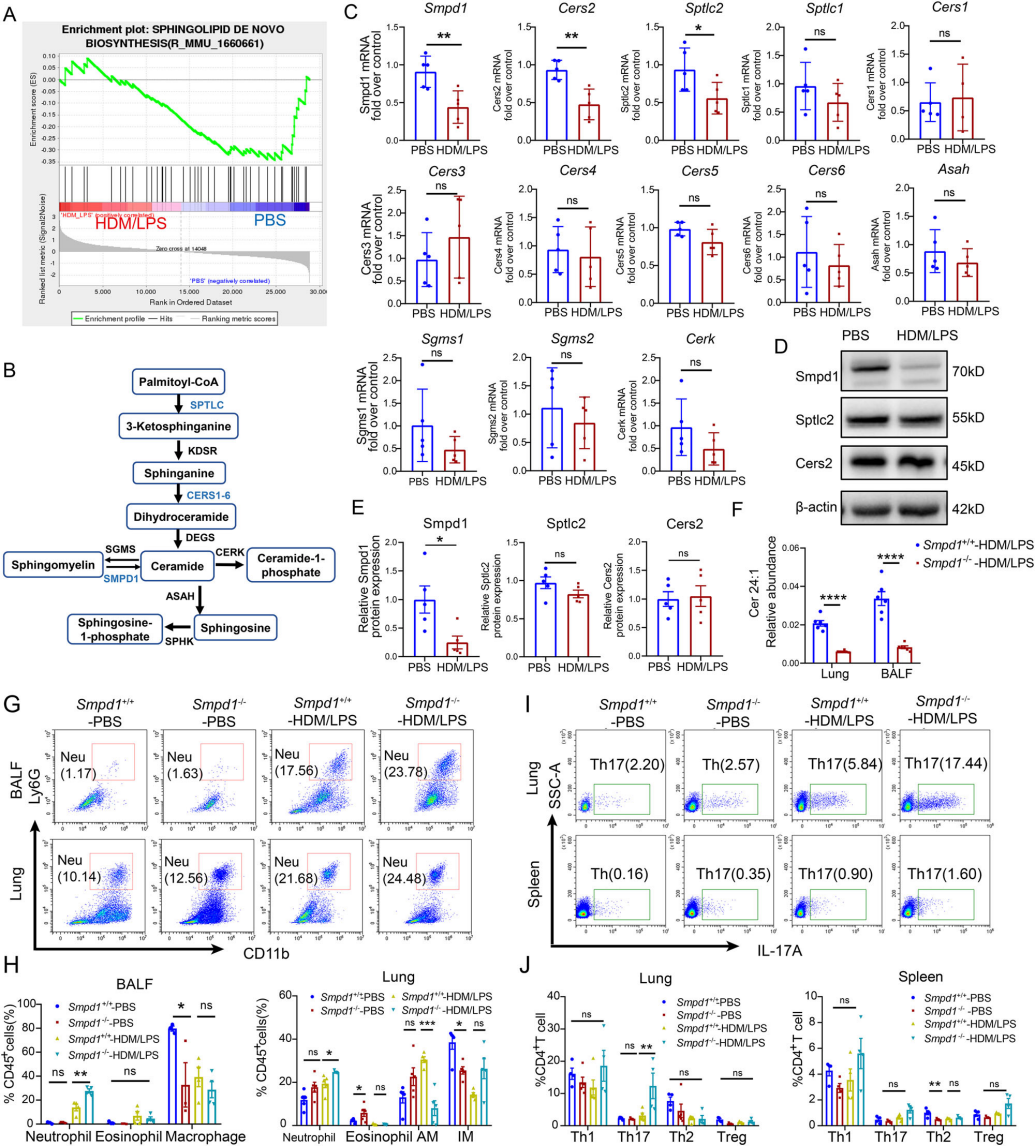
Sphingolipid biosynthesis pathway activity was inhibited in the lung tissue of HDM/LPS‐induced asthma mice. (A) GSEA of RNA‐seq data obtained from the lung tissues of PBS‐treated and HDM/LPS‐induced model mice revealed that the sphingolipid synthesis pathway was inhibited in HDM/LPS‐induced model mice (*n* = 6 mice/group). (B) Ceramide metabolic pathways. The diagram illustrates the metabolic pathways for ceramide synthesis, including the sphingomyelinase and de novo pathways. Abbreviations: SPTLC, serine palmitoyltransferase; KDSR, 3‐ketodihydrosphingosine reductase; CERS, ceramide synthase; DEGS, degenerative spermatocyte homolog; ASAH, N‐acylsphingosine amidohydrolase; SPHK, sphingosine kinase; SGMS, sphingomyelin synthase; SMPD1, sphingomyelin phosphodiesterase; CERK, ceramide kinase. (C) RT‒qPCR analysis of genes encoding enzymes involved in the *de novo* generation and sphingomyelinase pathways of ceramides in the lungs of HDM/LPS‐induced model mice compared with those of control mice. *Sptlc2*, *Cers2*, and *Smpd1* were downregulated in HDM/LPS‐induced model mice (*n* = 4–5 mice/group). (D,E) Representative immunoblot analysis of Sptlc2, Cers2, and Smpd1 protein expression in lung tissue from HDM/LPS‐induced model mice and controls. Smpd1 was downregulated in HDM/LPS‐induced model mice (*n* = 4 mice/group). (F) Relative abundance of Cer24:1 in the lungs of *Smpd1^−/−^* and *Smpd1^+/+^* HDM/LPS‐induced model mice (*n* = 6 mice/group). (G,H) Flow cytometric and statistical analyses of neutrophils, eosinophils, alveolar macrophages (AMs), and interstitial macrophages (IMs) in the BALF (*n* = 3 mice/group) and lungs (*n* = 6 mice/group). The proportions of all myeloid cells were analyzed by gating for Zombie Aqua^−^CD45^+^ cells. (I,J) Flow cytometry and statistical analysis of Th1, Th2, Th17, and Treg cells in the lungs and spleen (*n* = 6 mice/group). The proportions of all Th subgroups were analyzed by gating for Zombie Aqua^−^CD3^+^CD8^−^CD4^+^ T cells. The data are presented as the mean ± SEM. For comparisons between two groups, statistical analysis was performed using two‐tailed Student's *t* tests. For comparisons among four groups, statistical analysis was performed using one‐way ANOVA followed by Tukey's post hoc test (or Kruskal–Wallis test with Dunn's multiple comparisons test for nonnormally distributed data). ^*^ *p <* 0.05, ^**^ *p <* 0.01, ^***^ *p <* 0.001, ^****^ *p <* 0.0001; ns, not significant.

Gene set enrichment analysis (GSEA) of lung tissues from HDM/LPS‐induced mice revealed suppression of the sphingolipid biosynthesis pathway (Figure 4A). Ceramides are synthesized via *de novo* and sphingomyelin hydrolysis pathways (Figure 4B). While the mRNA levels of enzymes in these pathways (*Sptlc2*, *Cers2*, *Smpd1*) were reduced in NA lungs (Figure 4C), only the protein level of *Smpd1* (acid sphingomyelinase, the major producer of VLC‐Cers) was consistently decreased (Figure 4D,E).

To determine the pathological impact of reduced VLC‐Cer generation, we generated *Smpd1* ^−/−^ mice. These mice presented markedly lower Cer24:1 levels in the lungs and BALF (Figure 4F; Figure S5A). Compared with *Smpd1* ^+/+^ wild‐type (WT) controls, *Smpd1* ^−/−^ mice presented exacerbated airway resistance (Figure S5B) and increased plasma IL‐17A and IL‐22 levels after HDM/LPS induction (Figure S5C). HDM/LPS‐induced *Smpd1* ^−/−^ mice displayed aggravated pulmonary inflammation, as evidenced by increases in histological inflammation scores, the MPO‐positive areas (Figure S5D,E), and the numbers of neutrophils in BALF and lung (Figure 4G,H), without significant changes in the PAS staining results. Flow cytometric analysis revealed an increase in Th17 cells in the lungs of HDM/LPS‐induced *Smpd1* ^−/−^ mice and a reduction in Th2 cells in the spleens of PBS‐induced *Smpd1* ^−/−^ mice (Figure 4I,J).

Despite heightened inflammation, airway remodeling was not exacerbated in HDM/LPS‐induced *Smpd1* ^−/−^ mice, as shown by α‐SMA, fibronectin, and collagen staining (Figure S5F,G), suggesting that *Smpd1* influences remodeling via pathways beyond those associated with VLC‐Cer deficiency. Transcriptomic analyses confirmed the enrichment of Th17 differentiation pathways in the lungs of HDM/LPS‐induced *Smpd1* ^−/−^ mice (Figure S5H). Compared with wild‐type control T cells, *Smpd1* ^−^^/^^−^ T cells displayed increased Th17 differentiation in vitro (Figure S5I). Importantly, exogenous Cer24:1 partially rescued this phenotype and reduced Th17 differentiation in *Smpd1* ^−^^/^^−^ cells. Collectively, these findings suggest that *Smpd1* deficiency exacerbates Th17‐driven inflammation in the context of NA.

### 2.5 Cer24:1 Inhibits Th17 Cell Differentiation

**FIGURE 5:**
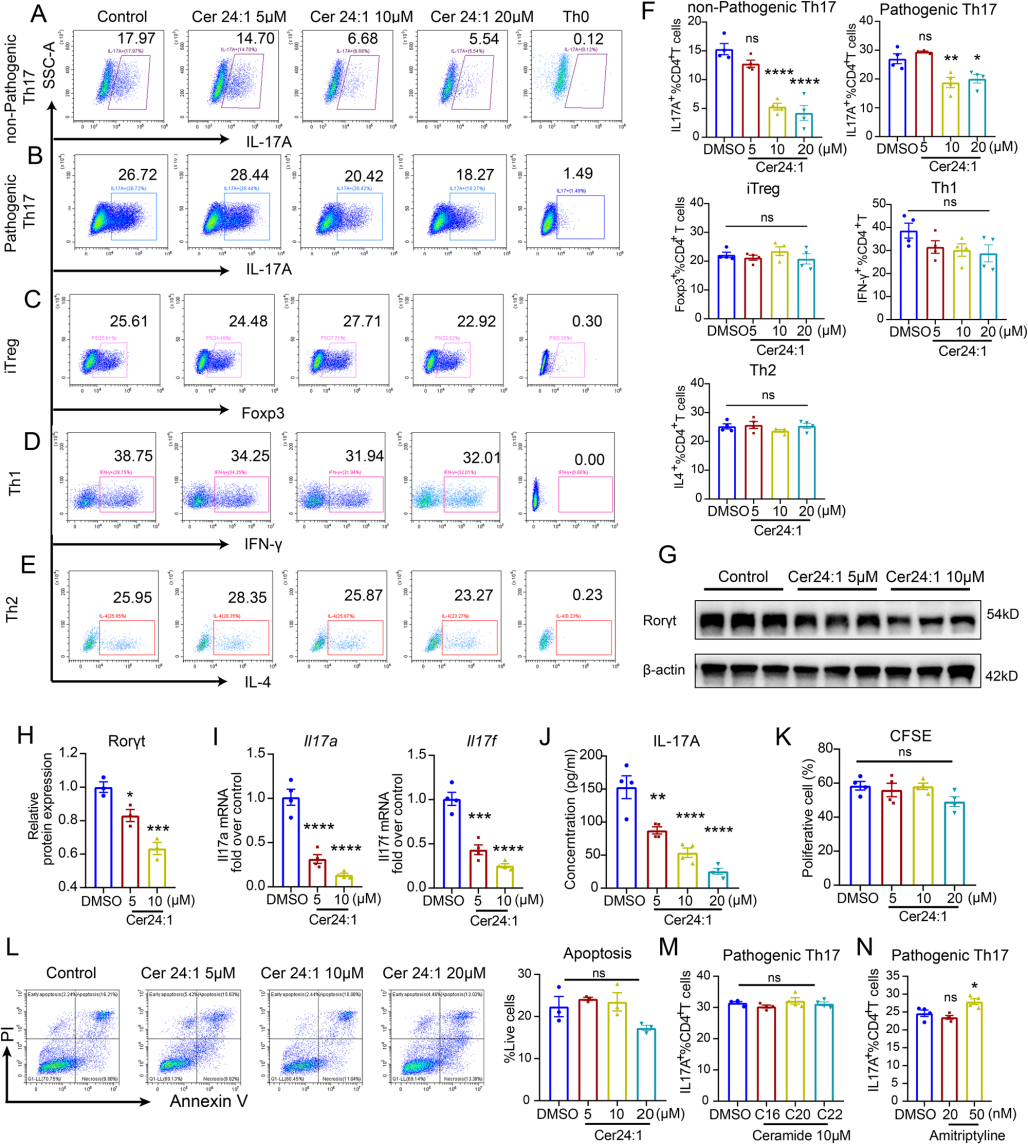
Cer24:1 inhibits Th17 differentiation in vitro. Naïve CD4^+^ T cells were polarized under Th1 (IL‐12 + anti‐IL‐4 + IL‐2), Th2 (IL‐4 + anti‐IFN‐γ + IL‐2), Th17 (IL‐6 + TGF‐β1 for nonpathogenic Th17 or IL‐6 + IL‐1β + IL‐23 + TGF‐β1 for pathogenic Th17) or iTreg (TGF‐β1 + IL‐2) culture conditions for 3–4 days, followed by restimulation with phorbol‐12‐myristate‐13‐acetate and ionomycin. (A–F) Flow cytometry analysis of Th1, Th2, Th17, and Treg cells under polarized conditions with Cer24:1 treatment. The proportions were gated on Zombie Aqua^−^CD4^+^ T cells for analysis (*n* = 4). (G,H) Representative immunoblot analysis of RORγt in pathogenic Th17 cells treated with Cer24:1. Densitometry was performed in ImageJ; target bands were normalized to β‐actin and expressed relative to control (set to 1). Data are mean ± SEM from three biological replicates. (I) Real‐time qPCR analysis of *Il17a* and *Il17f* mRNA expression in pathogenic Th17 cells treated with Cer24:1 for 3 days (*n* = 4). (J) The IL‐17A concentration in the cell culture supernatant was detected by ELISA. (K) CFSE‐labeled pathogenic Th17 cells were treated with Cer24:1 for 3 days and analyzed by flow cytometry (*n* = 4). (L) Representative flow cytometric analysis of the apoptosis of pathogenic Th17 cells treated with Cer24:1 for 3 days (*n* = 3). (M) The percentage of pathogenic Th17 cells after C16 (ceramide d18:1/16:0), C20 (ceramide d18:1/20:0), and C22 (ceramide d18:1/22:0) ceramide treatment for 3 days (*n* = 4). (N) The percentage of pathogenic Th17 cells treated with amitriptyline for 3 days (*n* = 4). The results are presented as the mean ± SEM. Statistical analysis was performed via one‐way ANOVA followed by Tukey's post hoc test (or Kruskal–Wallis test with Dunn's multiple comparisons test for nonnormally distributed data) for multi‐group comparisons. ^*^ *p <* 0.05, ^**^ *p <* 0.01, ^***^ *p <* 0.001, ^****^ *p <* 0.0001; ns, not significant.

To investigate the direct role of Cer24:1 in T‐cell differentiation, we generated in vitro differentiation models for pathogenic Th17 and nonpathogenic Th17 cells, as well as Th1, Th2, and iTreg cells. Cer24:1 selectively inhibited the differentiation of both Th17 subsets in a dose‐dependent manner (Figure 5A,B) without affecting Th1, Th2, or iTreg cell differentiation (Figure 5C–F). RT‒qPCR and Western blot analysis confirmed that 5 µm Cer24:1 significantly reduced the Rorγt protein level and decreased the *Il‐17a* and *Il‐17f* mRNA levels (Figure 5G,H,J). Consistently, IL‐17A secretion was also significantly decreased (Figure 5I). Importantly, Cer24:1 did not affect the apoptosis or proliferation of Th17 cells (Figure 5K,L), indicating a specific effect on lineage commitment rather than on cell viability or expansion. Notably, other ceramide species (C16: ceramide d18:1/16:0, C20: ceramide d18:1/20:0, C22: ceramide d18:1/22:0) did not suppress pathogenic or nonpathogenic Th17 cells (Figure 5M; Figure S5J), whereas amitriptyline hydrochloride (AMT), an acid sphingomyelinase inhibitor, significantly promoted Th17 differentiation (Figure 5N).

**FIGURE 6:**
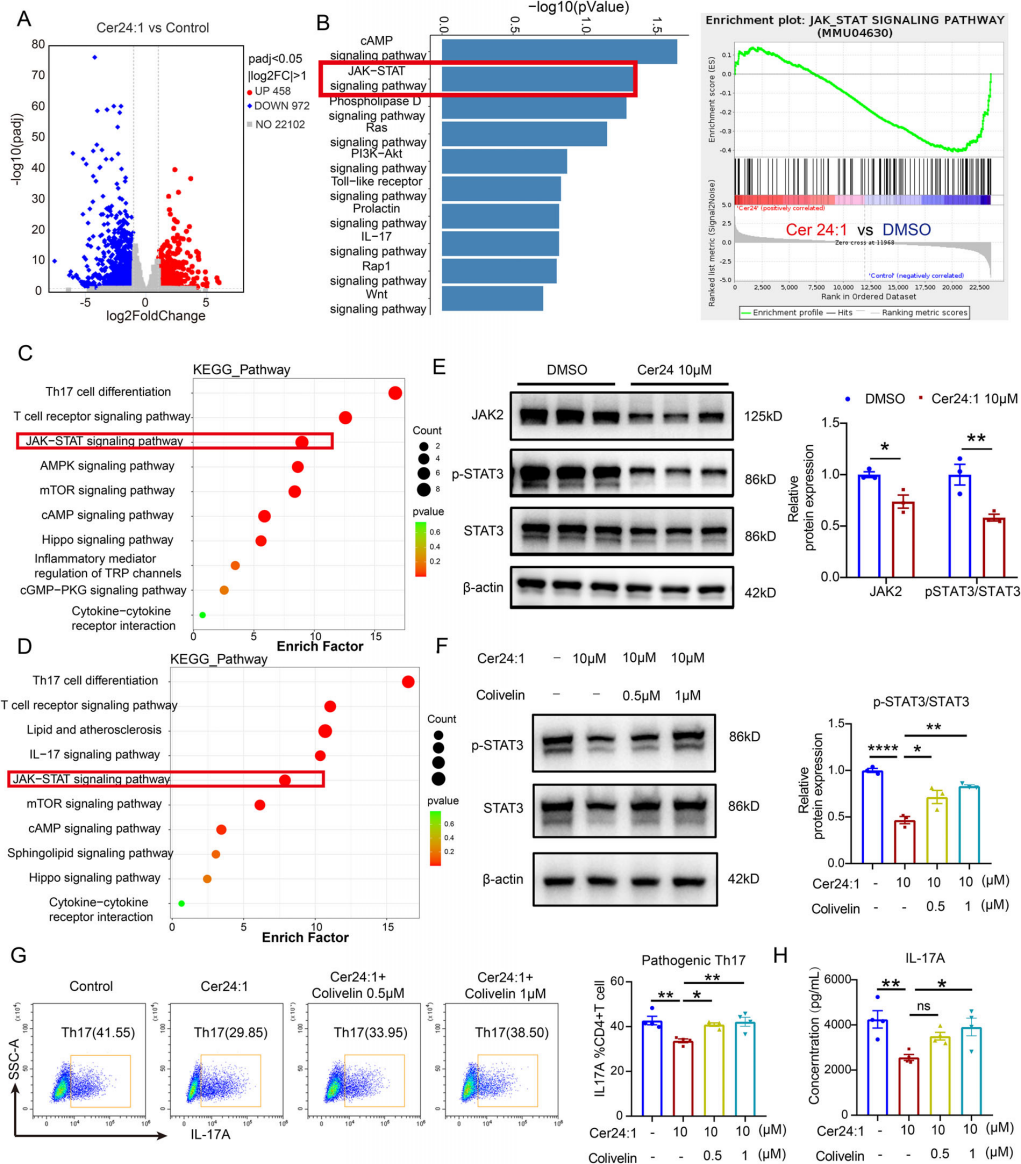
Cer24:1 inhibits the differentiation of pathogenic Th17 cells by inhibiting the JAK2–STAT3 signaling pathway. Naïve CD4^+^ T cells were polarized under Th17 conditions with DMSO or 10 µm Cer24:1 for 3 days. Pathogenic Th17 cells were collected for bulk RNA sequencing (A,B) or protein phosphorylation array (C,D). (A) Volcano plot showing the differentially expressed genes between the two groups according to the RNA‐seq results. (B) KEGG enrichment analysis and GSEA of the DEGs identified from the RNA‐seq results. The JAK–STAT pathway is highlighted in red. (C,D) KEGG enrichment analysis of the differentially phosphorylated proteins (C) and total proteins (D) detected in the protein phosphorylation array. The JAK–STAT pathway is highlighted in red. (E,F) Representative immunoblot analysis of phosphorylated JAK2 and STAT3 levels in Th17 cells treated with Cer24:1 or Cer24:1 combined with colivelin (a STAT3 activator). Densitometry was performed in ImageJ; target bands were normalized to β‐actin and expressed relative to control (set to 1). Data are mean ± SEM from three biological replicates. (G) Representative flow cytometric analysis of pathogenic Th17 cells treated with Cer24:1 or Cer24:1 in combination with colivelin (*n* = 3). (H) IL‐17A concentration in the cell culture supernatant of Th17 cells treated with Cer24:1 or Cer24:1 in combination with colivelin (*n* = 3). The data are presented as the mean ± SEM. Statistical analysis was performed via two‐tailed Student's *t* test (E) and one‐way ANOVA followed by Tukey's post hoc test (or Kruskal–Wallis test with Dunn's multiple comparisons test for non‐normally distributed data) (F–H). For RNA‐seq and phosphorylation array data (A–D), differentially expressed genes or phosphoproteins were defined using adjusted *p*‐value and log_2_ fold‐change thresholds as described in the RNA‐seq Analysis subsection. ^*^ *p <* 0.05, ^**^ *p <* 0.01, ^***^ *p <* 0.001, ^****^ *p <* 0.0001; ns, not significant.

Transcriptome analysis of Cer24:1‐treated Th17 cells revealed widespread downregulation of gene expression (Figure 6A), and KEGG enrichment analysis revealed that the JAK–STAT signaling pathway was among the pathways most significantly suppressed following Cer24:1 intervention (Figure 6B). Phosphoproteomic analysis confirmed that the JAK–STAT, Th17 differentiation, and T‐cell receptor pathways were enriched in the differentially phosphorylated proteins (Figure 6C,D). Western blot analysis verified that 10 µm Cer24:1 decreased phosphorylated STAT3 (p‐STAT3) and JAK2 protein levels (Figure 6E). Administration of the STAT3 agonist colivelin reversed these effects, restoring the levels of phosphorylated STAT3, Th17 differentiation, and IL‐17A secretion (Figure 6F–H), confirming that Cer24:1 inhibits Th17 polarization via suppression of the JAK2–STAT3 axis.

### 2.6 Cer24:1 Specifically Binds to the Prostaglandin E_2_ Receptor EP2 and Inhibits STAT3 Activation in Th17 Cells

**FIGURE 7:**
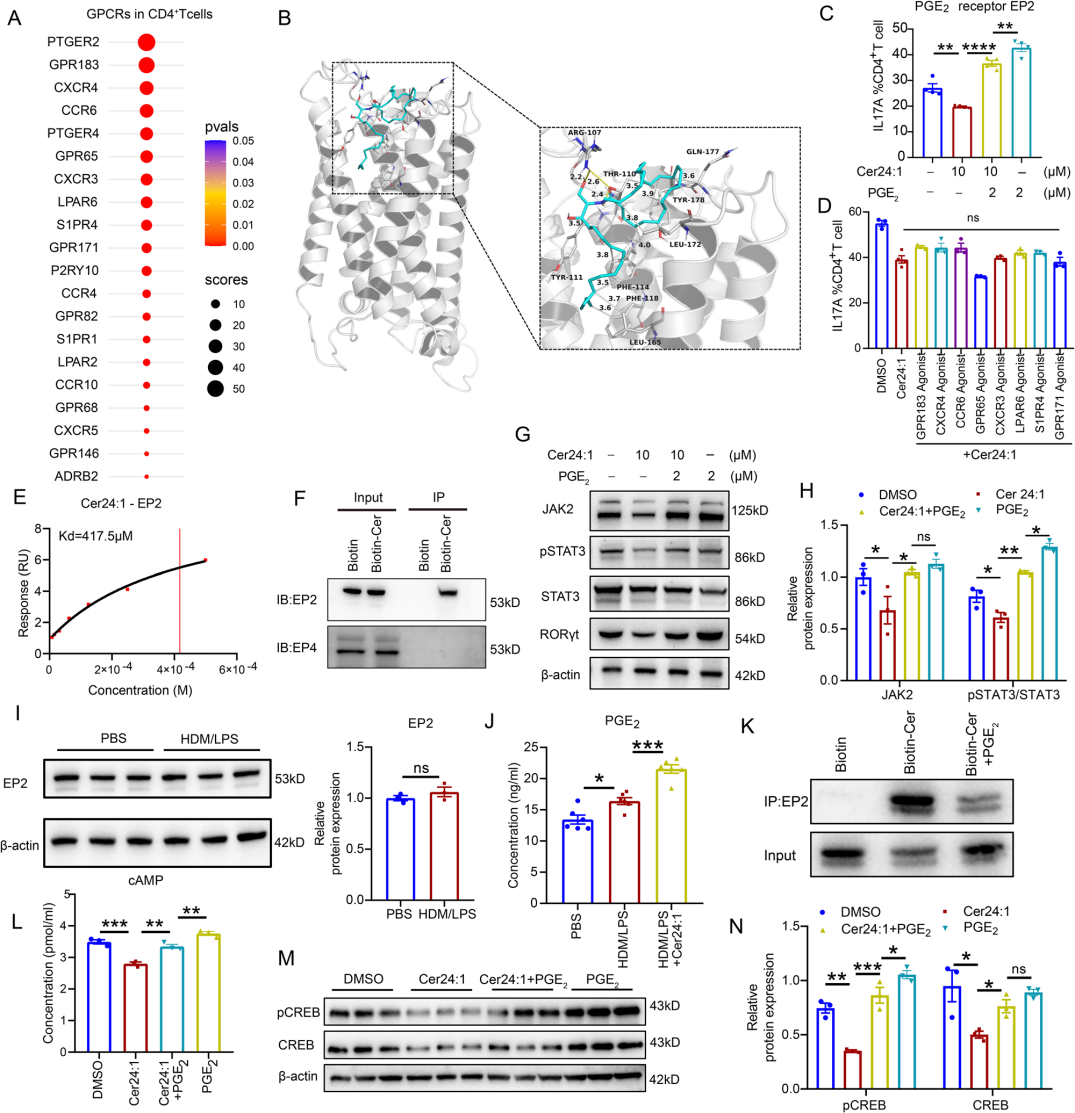
Cer24:1 inhibits activation of the STAT3 pathway by specifically binding to EP2 in Th17 cells. (A) Published single‐cell sequencing datasets (GSE135779, GSE142637, and GSE162577) revealed the top 20 G protein‐coupled receptor (GPCR) family genes expressed on CD4^+^ T cells. (B) Molecular docking prediction of the Cer24:1 binding site in the EP2 domain. The 3D structure of the EP2 protein is shown in silver. (C) Flow cytometric analysis of pathogenic Th17 cells treated with Cer24:1 or Cer24:1 in combination with PGE_2_ (a specific ligand of EP2) (*n* = 4). (D) Flow cytometric analysis of the percentage of pathogenic Th17 cells that were treated with Cer24:1 or Cer24:1 in combination with eight GPCR agonists (*n* = 4). (E) Line graphs showing the results of the SPR analysis of the binding between Cer24:1 and EP2. (F) Biotin‐ceramide pull‐down assay showing the interaction between Cer24:1 and EP2 in CD4^+^ T cells. The cell lysates were incubated with biotin‐ceramide or biotin, and the interacting proteins were isolated by streptavidin agarose pull‐down, followed by immunoblotting with anti‐EP2, anti‐EP4, and anti‐CXCR4 antibodies. (G,H) Representative immunoblot analysis of phosphorylated JAK2–STAT3 pathway proteins and RORγt in Th17 cells treated with Cer24:1 and PGE_2_. Densitometry was performed in ImageJ; target bands were normalized to β‐actin and expressed relative to control (set to 1). Data are mean ± SEM from three biological replicates. (I) Representative immunoblot analysis of EP2 expression in lung tissue from mice treated with PBS or HDM/LPS (*n* = 3 mice/group). (J) PGE_2_ concentration in plasma from HDM/LPS‐induced model mice and Cer24:1‐treated mice (*n* = 6 mice/group). (K) Biotin‐ceramide pull‐down assay showing that the interaction between Cer24:1 and EP2 in CD4^+^ T cells was suppressed by PGE_2_. (L) cAMP concentrations in Th17 cell lysates detected by ELISA. (M,N) Representative immunoblot analysis of phosphorylated CREB proteins and total proteins in Th17 cells treated with Cer24:1 and/or PGE_2_. The data are presented as the mean ± SEM. Statistical analysis was performed via two‐tailed Student's *t* test (I) and one‐way ANOVA followed by Tukey's post hoc test (or Kruskal–Wallis test with Dunn's multiple comparisons test) (B,C,H,J,L,N). ^*^ *p <* 0.05, ^**^ *p <* 0.01, ^***^ *p <* 0.001, ^****^ *p <* 0.0001; ns, not significant.

The hydrophilic sphingosine head group and very‐long hydrophobic chain of Cer24:1 limit its ability to cross the cell membrane via simple lipophilic diffusion, suggesting potential action via membrane receptors. To investigate whether ceramides directly interact with specific cell membrane receptors, such as GPCRs, we analyzed multiple available single‐cell sequencing datasets and focused on 764 GPCRs expressed by CD4^+^ T cells. The top 20 genes encoding GPCRs (PTGER2, GPR183, CXCR4, CCR6, PTGER4, GPR65, CXCR3, LPAR6, S1PR4, GPR171, P2RY10, CCR4, GPR82, S1PR1, LPAR2, CCR10, GPR68, CXCR5, GPR146, and ADRB2) were ranked according to their relative abundance on CD4^+^ T cells (Figure 7A; Figure S6A). We performed structure‐guided molecular docking simulations between Cer24:1 and the top 10 GPCR proteins and calculated the corresponding binding energies. The predictions revealed that all these GPCRs exhibited a certain potential to bind Cer24:1, with PTGER2 binding being the most likely (binding affinity: ‐7.064 kcal/mol; Table S2). Further molecular docking and molecular dynamics simulations predicted that Cer24:1 binds to EP2 via ARG‐107, TYR‐111, and SER‐169 (Figure 7B; Figure S6C,D). In functional assays, among the agonists or ligands of these GPCRs tested during the Cer24:1‐mediated inhibition of Th17 differentiation, PGE_2_ (an EP2 ligand) reversed the inhibitory effect most effectively (Figure 7C,D). The direct interaction between Cer24:1 and EP2 was validated by surface plasmon resonance (SPR) (Figure 7E; Figure S6B). Additionally, ceramide–biotin pull‐down assays confirmed that Cer24:1 selectively binds to EP2 but not to EP4 (Figure 7F). Western blot analysis confirmed that PGE_2_ supplementation reversed the Cer24:1‐induced decreases in JAK2 and STAT3 phosphorylation and Rorγt expression in Th17 cells (Figure 7G,H). While EP2 expression in lung tissues was unchanged between HDM/LPS‐induced model mice and control mice (Figure 7I), NA model mice had elevated plasma PGE_2_ levels, which further increased after Cer24:1 treatment (Figure 7J). In addition, biotin‐ceramide pull‐down assays revealed that the interaction between Cer24:1 and EP2 in CD4^+^ T cells was attenuated by PGE_2_ (Figure 7K), further supporting competitive EP2 binding. In vitro, EP2 inhibition with AH6809 reduced Th17 differentiation (Figure S6E), supporting the hypothesis that Cer24:1 antagonizes PGE_2_ by competitively binding to EP2, thereby attenuating downstream JAK2–STAT3 activation (Figure S7). Notably, as a GPCR, EP2 signaling activates the cAMP pathway. KEGG enrichment analysis revealed significant suppression of cAMP‐related pathways in Cer24:1‐treated Th17 cells (Figure 6B), which is consistent with the canonical signaling mechanism of EP2. Furthermore, Cer24:1 treatment reduced the intracellular cAMP level and the phosphorylation of cAMP‐response element binding protein (CREB) in Th17 cells, as shown by ELISA and Western blotting (Figure 7L–N), whereas PGE_2_ reversed both of these changes, confirming the involvement of EP2–cAMP–CREB in Th17 regulation.

### 2.7 PGE_2_ Abrogates the Alleviating Effect of Cer24:1 in NA Model Mice

**FIGURE 8:**
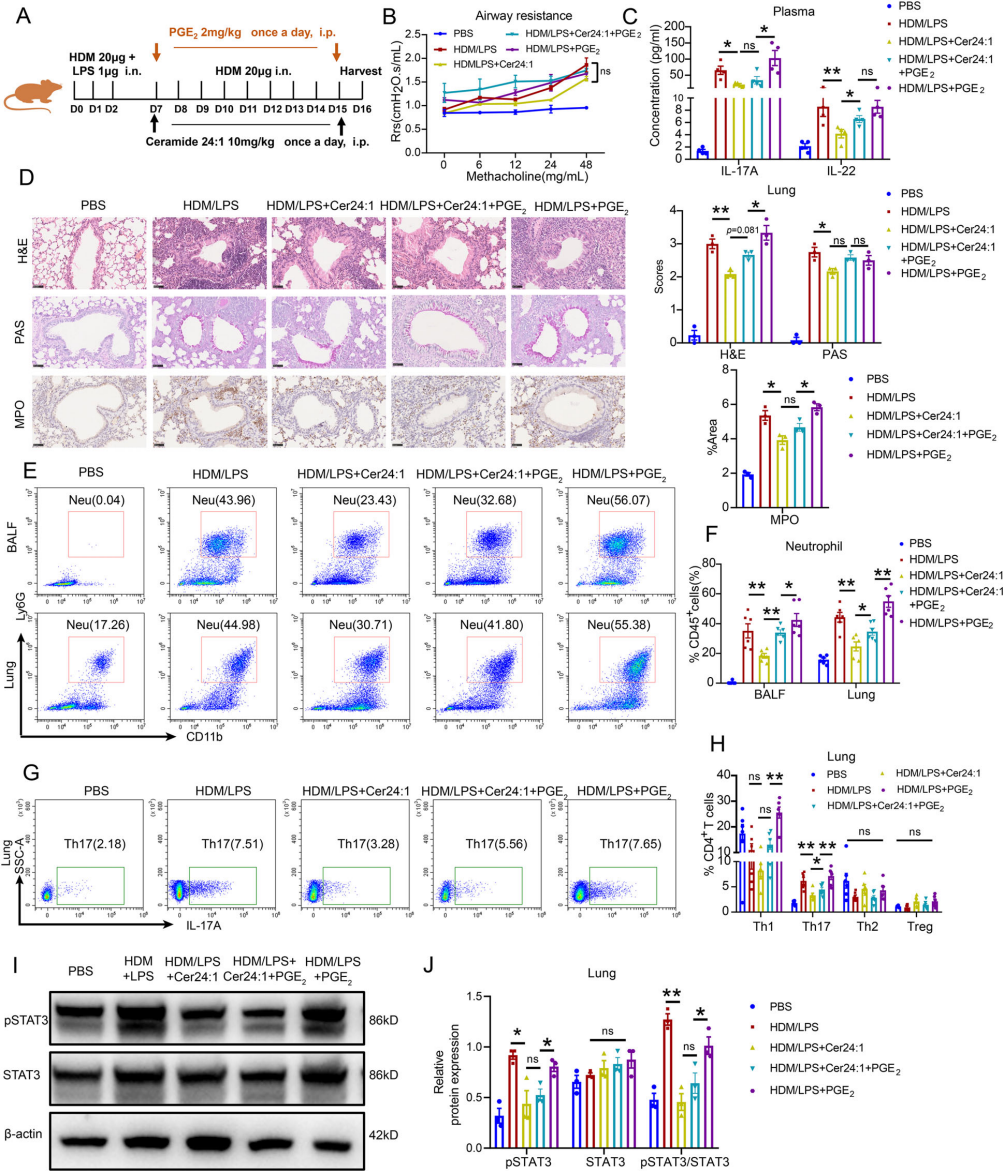
PGE_2_ significantly abrogated the therapeutic effect of Cer24:1 on HDM/LPS‐induced asthma in mice. All groups except the control group were induced with HDM/LPS to establish the asthma model. (A) Illustration of the experimental design. (B) Airway resistance in response to methacholine treatment. (C) IL‐17A and IL‐22 concentrations in plasma and BALF were detected by CBA. (D) Representative H&E, PAS, and immunohistochemical staining of MPO in lung sections, along with a histogram of the pathological score. Scale bar = 50 µm (*n* = 3 mice/group). (E–F) Flow cytometric analysis of neutrophils, eosinophils, alveolar macrophages (AMs), and interstitial macrophages (IMs) in BALF and lung tissue (*n* = 5–6 mice/group). The proportion of all myeloid cells was analyzed by gating on Zombie Aqua^−^CD45^+^ cells. (G,H) Flow cytometric analysis of Th1, Th2, Th17, and Treg cells in lung tissue (*n* = 6 mice/group). The proportions of all T‐cell subsets were analyzed by gating on Zombie Aqua^−^CD3^+^CD8^−^CD4^+^ T cells. (I,J) Representative immunoblot analysis of phosphorylated JAK2–STAT3 pathway proteins in lung tissue from PBS‐treated mice and HDM/LPS‐induced mice treated with DMSO, Cer24:1, PGE_2,_ or Cer24:1 combined with PGE_2_. Densitometry was performed in ImageJ; target bands were normalized to β‐actin and expressed relative to control (set to 1). Data are mean ± SEM from three biological replicates. The data are presented as the mean ± SEM. Statistical analysis was performed via one‐way ANOVA followed by Tukey's post hoc test (or Kruskal–Wallis test with Dunn's multiple comparisons test for nonnormally distributed data). ^*^ *p <* 0.05, ^**^ *p <* 0.01, ^***^ *p <* 0.001, ^****^ *p <* 0.0001; ns, not significant.

To explore the interaction between Cer24:1 and PGE_2_ in NA model mice, we administered either exogenous PGE_2_ or vehicle control (DMSO) to Cer24:1‐treated NA model mice (Figure 8A). Although the combination of PGE_2_ and Cer24:1 did not further exacerbate airway resistance compared with Cer24:1 treatment alone (Figure 8B), it significantly elevated plasma IL‐22 levels (Figure 8C). Compared with Cer24:1 + PGE_2_ treatment, PGE_2_ alone induced greater lung inflammation and neutrophil infiltration, suggesting partial retention of the protective effects of Cer24:1 (Figure 8D). Flow cytometry revealed that PGE_2_ reversed the Cer24:1‐induced reduction in neutrophils and Th17 cells in both BALF and lung tissue (Figure 8E–H). Western blot analysis revealed that Cer24:1 suppressed pSTAT3 levels, and compared with PGE_2_ treatment alone, Cer24:1 + PGE_2_ treatment also significantly decreased p‐STAT3 expression in lung tissue (Figure 8I,J). Notably, PGE_2_ did not further aggravate airway remodeling, as collagen deposition and α‐SMA and fibronectin levels were comparable across the groups (Figure S8A–C), but PGE_2_ did increase the plasma concentrations of MMP‐9. The plasma concentrations of TIMP‐1 remained unaffected by PGE_2_ treatment (Figure S8D,E). These results indicate that PGE_2_ selectively counteracts the anti‐inflammatory effects of Cer24:1 by promoting Th17 cell expansion and STAT3 activation.

## 3 Discussion

Our study demonstrates thatceramide Cer24:1 is significantly decreased in the context of neutrophilic asthma and functions as a critical endogenous regulator that constrains pathogenic Th17 responses. Through a translational approach combining human cohort analyses and murine models, we consistently observed a reduction in the level of Cer24:1 in multiple sample types. Administration of Cer24:1 markedly attenuated neutrophilic inflammation and key pathological features of asthma in mice, whereas Cer24:1 deficiency exacerbated disease severity. These findings consistently implicate Cer24:1 as an endogenous regulator of NA pathology with protective functions.

Our findings extend the current understanding of lipid mediators in asthma beyond classical eicosanoids and specialized pro‐resolving mediators. Although prior research has focused predominantly on arachidonic acid‐derived lipids [23, 24, 25], we revealed a sphingolipid‐centric pathway that directly modulates T‐cell polarization. Unlike conventional proinflammatory lipids such as certain prostaglandins and leukotrienes, Cer24:1 acts as an immunomodulatory mediator that specifically affects T‐cell polarization. Its interaction with EP2 suggests cross‐talk with established lipid signaling networks, particularly the PGE_2_‐EP2/EP4 axis, which is known to influence both Th17 differentiation and neutrophil function. We propose that Cer24:1 may serve as an endogenous ligand that competes with or modulates prostaglandin‐mediated responses, thereby fine‐tuning the inflammatory microenvironment in NA patients. This pathway represents a promising target for therapeutic intervention, especially in patients with steroid‐resistant endotypes for whom conventional therapies have proven ineffective.

In our cohort, Cer24:1 was consistently decreased in EBC, plasma, and sputum samples from NA patients, and this reduction correlated with more severe disease and poorer asthma control, suggesting a distinct metabolic signature. Our broader sampling strategy and inclusion of patients with chronic persistent asthma likely captured a more representative metabolic state. Moreover, ceramide levels may fluctuate across disease stages; ongoing studies involving sample collection during acute exacerbations will help clarify potential compensatory changes. Interestingly, James et al. reported elevated BALF levels of ceramides, including C20, C26, and C26:1 in severe asthma patients [26]. Such differences may reflect methodological and cohort‐level factors, including sample source (BALF vs. EBC/plasma/sputum), internal phenotype composition (proportions of severe asthma and NA patients), and sample size. Taken together, both studies support a role for sphingolipid metabolism in the pathobiology of asthma. We observed that Cer24:1 reduction is particularly pronounced in NA and inversely correlated with Th17 cells, while also showing some decrease in non‐NA asthma. Whether this reduction has broader significance and impact requires further validation in larger, deeply phenotyped cohorts.

Consistent with endotype specificity, we observed increased ceramide species in HDM‐induced eosinophilic asthma mice relative to controls and NA model mice, aligning with the trend reported by James et al. Mechanistically, we found that the expression of Smpd1 (a key enzyme in sphingomyelin hydrolysis) was downregulated in the lungs of NA model mice and that *Smpd1* deficiency reduced the levels of VLC‐ceramides, with Cer24:1 among the most altered species, suggesting that SMPD1 is an upstream contributor to VLC‐ceramide homeostasis. *Smpd1* knockout exacerbated Th17‐driven inflammation, which is consistent with the findings of previous studies that linked impaired ceramide synthesis to immune dysregulation [27]. Moreover, exogenous Cer24:1 partially rescued the increase in Th17 polarization observed in *Smpd1* ^−/−^ CD4^+^ T cells, supporting a functional link between the SMPD1–Cer24:1 axis and Th17 cell pathogenicity.

Although the regulatory mechanisms of ceramides remain incompletely understood, emerging evidence highlights the importance of chain length‐dependent functionality. For instance, C24 ceramide (C24‐Cer) has been identified as a biomarker in gallbladder cancer and promotes cancer cell proliferation [28]. Long‐chain ceramides (LC‐Cers) are generally associated with cellular damage and inflammation [29], whereas very‐long‐chain ceramides (VLC‐Cers) contribute to membrane stability and barrier function [30], and may have anti‐inflammatory or cytoprotective effects [31]. Our study highlights important chain length‐dependent differences in ceramide immunomodulation: most VLC‐Cers, particularly Cer24:1, were inversely correlated with Th17 cell proportions and sputum neutrophil counts, whereas most LC‐Cers did not show significant associations. Although one prior study showed that intranasal Cer16:0 promotes neutrophil recruitment in HDM‐induced asthmatic mice [26], our results indicate that Cer24:1 exerts opposing, anti‐inflammatory effects.

Functionally, Cer24:1 administration reduced both Th17 cell and neutrophil infiltration in the lungs. Th17 cells promote neutrophil recruitment and activation via proinflammatory cytokine secretion [32]. Subsequent in vitro and in vivo experiments demonstrated that Cer24:1 modulates Th17 cells and attenuates neutrophilic inflammation. Mechanistically, Cer24:1 inhibited the differentiation of both pathogenic and nonpathogenic Th17 cells without affecting Th1, Th2, or anti‐inflammatory iTreg cells. Pathogenic Th17 cells dominate in severe steroid‐resistant asthma and drive IL‐17‐related inflammatory disorders [33]. Th17 cell differentiation depends on multiple signaling pathways, including the IL‐6–JAK2–STAT3 axis that is critical for CD4^+^ T‐cell fate determination [34, 35, 36]. In our study, we found that Cer24:1 inhibits JAK2–STAT3 signaling, reduces STAT3 phosphorylation, and downregulates RORγt expression. This selective suppression of the JAK2–STAT3–RORγt axis clarifies its specific effect on Th17 cells and positions it as a potential therapeutic target for Th17‐dominant, steroid‐resistant asthma.

Ceramides such as Cer24:1 anchor to the cell membrane and participate in lipid raft formation alongside proteins, thereby regulating signal transduction [37]. Recent studies have indicated that sphingolipids and glycerophospholipids frequently bind to GPCRs: lysophosphatidylcholine (LPC) binds to GPR40, GPR55, and GPR119 [38]; sphingosine‐1‐phosphate (S1P) interacts with S1PR [39]; and Cer16:0 also interacts with formyl peptide receptor 2 (FPR2) [40]. Here, we identify EP2 as a functional receptor for Cer24:1. EP2–PGE_2_ signaling mediates JAK2–STAT3 activation [41], and PGE_2_ promotes Th17 differentiation via the cAMP pathway and EP2/EP4 receptor signaling [42]. Clinically, sputum PGE_2_ levels are elevated in severe asthma patients [43] and are positively correlated with neutrophilic airway inflammation [44]. We propose that Cer24:1 competes with PGE_2_ for EP2 binding, thereby inhibiting downstream STAT3 activation and Th17 differentiation. Using surface plasmon resonance together with biotin–ceramide pull‐down assays, we provide evidence that Cer24:1 can bind to EP2. Nonetheless, the extent to which EP2 recognizes other ceramide species has not been systematically defined. The very‐long acyl chain and unsaturation may favor selective bioactivity by promoting partitioning into specific membrane microdomains or distinct membrane presentation, thereby enhancing effective engagement with membrane‐associated signaling machinery [40].

The inhibitory effect of Cer24:1 was most pronounced in the Th17 compartment, as Cer24:1 consistently attenuated Th17 differentiation and Th17‐associated inflammation without producing comparable changes in Th1, Th2, or Treg cells under the conditions tested. Mechanistically, our data support EP2–JAK2–STAT3 signaling as a key node targeted by Cer24:1, which may be particularly consequential for Th17 programs that are strongly reinforced by STAT3‐centered cytokine cues. Together, these findings reveal a chain length‐dependent immunomodulatory function of very‐long‐chain ceramides and suggest that Cer24:1 may be therapeutically leveraged in Th17‐driven inflammatory diseases. We further propose that the preferential efficacy of Cer24:1 in neutrophilic asthma reflects the greater dependence of pathogenic Th17 differentiation and maintenance on STAT3‐linked pathways, whereas eosinophilic asthma is predominantly driven by Th2‐type circuitry that may be relatively insensitive to modulation of the EP2–STAT3 axis. Although metabolite‐based therapies remain underexplored in asthma, they show promise in other respiratory conditions. For example, butyrate reduces ILC2‐mediated inflammation in COPD [45, 46], and S1P has been proposed as a protective agent for lung and cardiac vasculature during SARS‐CoV‐2 infection [47]. Our findings suggest that Cer24:1 supplementation represents a viable strategy to suppress Th17‐driven inflammation in patients with NA.

In conclusion, Cer24:1 levels are reduced in NA patients and are negatively correlated with the Th17 cell proportions and neutrophil counts. Mechanistically, Cer24:1 binds EP2 and inhibits the EP2–JAK2–STAT3–RORγt signaling pathway, thereby suppressing Th17 differentiation and ameliorating airway inflammation and remodeling. These results highlight Cer24:1 as a specialized pro‐resolving mediator and potential metabolic therapeutic agent for treating neutrophilic asthma.

## 4 Materials and Methods

### 4.1 Human Participants

Asthma patients and healthy controls were recruited from Peking University Third Hospital. All participants provided written informed consent. This study was approved by the hospital's Ethics Committee (Approval No. LM2023295). The inclusion criteria required participants to have a confirmed diagnosis of asthma in accordance with the GINA guidelines. Pregnant women and patients with acute asthma exacerbations within the past 4 weeks; coexisting respiratory diseases; severe cardiovascular, hepatic or renal diseases; autoimmune diseases; metabolic diseases; malignant diseases; and immunodeficiency or other major medical illnesses were excluded. Patients who had used systemic steroids or antibiotics within 2 weeks prior to enrollment were also excluded.

Eligible participants underwent sputum induction to assess cell composition and perform classification analysis. A total of 28 asthma participants did not provide induced sputum and were therefore excluded from sputum‐based inflammatory phenotyping and related analyses. Sputum inflammatory phenotypes were defined using established cutoffs (neutrophils ≥ 61% for neutrophilic asthma; eosinophils ≥ 3% for eosinophilic asthma; both elevated for mixed granulocytic asthma; and neither elevated for paucigranulocytic asthma). Patients were classified into four subtypes: NA (*n* = 85), eosinophilic asthma (*n* = 39), mixed granulocytic asthma (*n* = 13), and paucigranulocytic asthma (*n* = 8). A total of 53 healthy volunteers (HCs) were also recruited from Peking University Third Hospital and were required to meet specific criteria, including normal lung function and the absence of asthma‐related symptoms, such as wheezing. The age, sex, and BMI of each of the healthy volunteers were matched to those of the asthma patients to ensure comparability. Not all participants provided all the biospecimens; sample availability varied by specimen type, and the exact numbers used for each analysis are indicated in the corresponding figure legends.

### 4.2 Animals

C57BL/6J wild‐type mice were obtained from the Laboratory Animal Science Department at Peking University, and *Smpd1*‐KO mice were provided by Jiangsu Jicui Pharmaceutical. Wild‐type *Smpd1* ^+/+^ littermates were used as controls. All the mice were randomly assigned to experimental groups and housed under standard specific pathogen‐free (SPF) laboratory conditions, with unrestricted access to food and water. The housing conditions included temperatures ranging from 21°C–24°C, humidity levels ranging from 40% to 70% and a controlled light cycle from 07:00–19:00. All animal experiments were approved by the Peking University Medical School Animal Care and Use Committee (Approval No. LA2022692).

The NA mouse model was established following previously described protocols [20, 21, 22]. In brief, 6‐ to 8‐week‐old mice were sensitized via intranasal (i.n.) administration of 20 µg of HDM (Cat# XPB82D3A25; Greer, London, USA) with or without 1 µg of LPS (O111:B4; Cat# L4391; Sigma–Aldrich, St. Louis, MO, USA) dissolved in 20 µL of PBS on days 0, 1, and 2. From day 7 to 15, the mice were challenged daily with 20 µg of HDM alone for nine consecutive days. Final analyses were performed one day after the last HDM exposure. For the HDM‐induced eosinophilic asthma model, 6‐ to 8‐week‐old mice were sensitized via intranasal (i.n.) administration of 1 µg of HDM dissolved in 20 µL of PBS on day 0. From day 7 to 11, the mice were challenged daily with 10 µg of HDM alone for 5 consecutive days.

For Cer24:1 administration, mice received daily intraperitoneal injections (10 mg/kg body weight; Cayman Chemical #62530, Michigan, USA) or DMSO vehicle from day 7 to day 15 (NA model) or day 7 to day 11 (EA model), which was administered 2 h prior to each challenge.

For PGE_2_ administration, mice received daily intraperitoneal injections of either PGE_2_ (2 mg/kg body weight; Cat# HY‐101952; MedChemExpress) or DMSO vehicle from day 7 to 15 in the NA model, concurrent with Cer24:1 treatment.

### 4.3 Sample Collection and Standardized Processing

EBC was collected using the TURBO‐DECCS system (Medivac PARMA, Italy) according to the manufacturer's guidelines during 15 min of tidal breathing. Participants rinsed their mouths with water prior to sample collection to minimize saliva contamination. The samples were immediately centrifuged (3000 × g, 10 min, 4°C) to remove particulate matter. The supernatant was aliquoted and stored at −80°C until lipid extraction. To account for dilution effects, EBC density was determined by gravimetric analysis, and metabolite concentrations were normalized to the density‐adjusted values.

Sputum induction was performed using inhaled hypertonic saline (3%) for 15 min. Sputum samples were weighed (wet mass) and homogenized with four volumes of 0.1% dithiothreitol (DTT)/PBS solution (w/v) by vortexing (30 s) and incubation (15 min, RT). After centrifugation (300 × g, 10 min, 4°C), the supernatant was collected and stored at ‐80°C. The total protein concentration (Bradford assay) was used for normalization to account for variability in mucus dilution.

Peripheral blood was collected in K_2_EDTA tubes and processed within 30 min of collection. The samples were centrifuged at 1500 × g for 15 min to obtain platelet‐poor plasma, after which the plasma was then collected and stored at −80°C.

BALF was collected by cannulating the trachea and flushing the lungs three times with 0.8 mL of ice‐cold PBS. After centrifugation (300 × g, 10 min, 4°C), the supernatants were aliquoted and stored at −80°C. BALF metabolite concentrations were normalized to the recovery volume.

Lung tissues were rinsed with ice‐cold PBS to remove residual blood, blotted dry on sterile filter paper, and immediately snap‐frozen in liquid nitrogen. The lung metabolite concentrations were normalized to the tissue weight (mg).

### 4.4 Cell Culture and Differentiation

Naïve CD4^+^ T cells were isolated from the spleens and lymph nodes of C57BL/6 wild‐type mice using a naïve CD4^+^ T‐cell isolation kit (Cat# 130‐104‐453; Miltenyi Biotec). The purified CD4^+^ T cells were cultured in serum‐free lymphocyte medium (Cat# 88‐581‐CM; Corning) supplemented with 10% fetal calf serum (Cat# 11560636; Gibco) and 1% penicillin/streptomycin (Cat# 15070063; Gibco). The cells were stimulated with 5 µg/mL anti‐CD3 (Cat# 100340; BioLegend) and 5 µg/mL anti‐CD28 (Cat# 102116, BioLegend) antibodies and polarized with various cytokine cocktails. Cer24:1, ceramide d18:1/16:0 (C16), ceramide d18:1/20:0 (C20), ceramide d18:1/22:0 (C22), or a DMSO vehicle control was added at the start of polarization and maintained throughout the 72 h differentiation period without washout. Cer24:1 was prepared as a 10 mm stock solution in DMSO and briefly warmed (40°C–50°C) and sonicated to ensure complete dissolution before dilution with culture medium. At the end of differentiation, the cells were harvested and analyzed by flow cytometry to determine the frequencies of the indicated T‐cell subsets.

### 4.5 Assessment of Lung Function

Lung function was evaluated using the flexiVent system (Scireq, Montreal, Canada). Key parameters, including the total resistance of the respiratory system (Rrs), were measured. Airway responsiveness was assessed through the forced oscillation technique, which involves nebulizing increasing concentrations of methacholine (0, 6, 12, 24, and 48 mg/mL) in PBS via an ultrasonic nebulizer.

### 4.6 Single‐Cell Dissociation of Lung and Spleen Tissues

After the right lung was dissected, the tissue was incubated at 37°C for 40 min in an oscillating incubator with collagenase type IV and deoxyribonuclease I (Worthington Biochem, Lakewood, NJ, USA). After digestion, the tissue was filtered through a 40 µm mesh to obtain a single‐cell suspension, followed by red blood cell lysis (Cat# R1010, Solarbio). The spleen was processed using a similar procedure. The isolated cells were then washed twice with PBS and resuspended to a final concentration of 1 × 10^7^ cells/ml. The cells were subsequently stained and analyzed using flow cytometry.

### 4.7 Flow Cytometry

The cells were resuspended in PBS and stained with the Zombie Aqua Fixable Viability Kit (Cat#42310; BioLegend) to differentiate live cell from dead cells. After viability staining, surface markers were applied to the cells following the manufacturer's instructions. For intracellular transcription factor staining, the cells were fixed and permeabilized with eBioscience Fix/Perm solution (Cat#00‐5523‐00; Invitrogen) and then stained with FOXP3 according to the provided protocol. Flow cytometry analysis was conducted using a CytoFLEX cell analyzer.

For cytokine staining, the cells were stimulated for 5 h in the presence of the Cell Activation Cocktail (Cat#423301; BioLegend) and brefeldin A solution (Cat#420601; BioLegend). After stimulation, surface marker staining was carried out as previously described. Intracellular cytokine staining was then performed by fixing and permeabilizing the cells, followed by staining for IL‐17A, IFN‐γ, or IL‐4. The analysis was conducted using a CytoFLEX cell analyzer, and the data were processed using FlowJo 10.

For tissue flow cytometry, neutrophils were identified as CD45^+^Ly6G^+^CD11b^+^, eosinophils as CD45^+^CD11C^−^SiglecF^+^, alveolar macrophages as CD45^+^CD11C^+^SiglecF^+^, and interstitial macrophages as CD45^+^CD11C^−^SiglecF^−^Ly6G^−^CD11b^+^. Th1 cells were considered CD3^+^CD4^+^CD8^−^IFNγ^+^, Th2 cells were considered CD3^+^CD4^+^CD8^−^IL4^+^, Th17 cells were considered CD3^+^CD4^+^CD8^−^IL‐17A^+^, and Treg cells were considered CD3^+^CD4^+^CD8^−^Foxp3^+^.

### 4.8 Cell Apoptosis Assay

The apoptosis of Th17 cells was analyzed using an Annexin V‐FITC/PI Apoptosis Detection Kit (Cat# KGA106; Keygen Bio) according to the manufacturer's protocol.

### 4.9 CFSE‐Based T‐Cell Proliferation Assay

Purified naïve CD4^+^ T cells were labeled with 2.5 µm carboxyfluorescein succinimidyl ester (CFSE; Cat# C34554; Thermo Fisher) for 10 min at 37°C, followed by quenching with complete RPMI‐1640 medium. Labeled cells (1 × 10^5^ cells/well) were cultured in the presence of anti‐CD3/CD28 stimulation for 72 h. Cells were then harvested and analyzed by flow cytometry. Proliferation was assessed based on CFSE dilution using FlowJo software.

### 4.10 Quantification of Plasma Proinflammatory Cytokine Levels

A multiplexed microbead‐based immunoassay system (LEGENDPlex; BioLegend) was used to quantify proinflammatory cytokines, including IL‐17A, IL‐17F, IL‐22, and 12 additional cytokines, according to the manufacturer's guidelines. A LEGENDPlex MU Th Cytokine Panel (12‐plex) (Cat#741044; BioLegend) was used for this analysis. Data acquisition was performed using a CytoFLEX S flow cytometer (Beckman Coulter), and the results were analyzed with proprietary software provided by BioLegend.

### 4.11 ELISA

The levels of IL‐17A (Cat#M1700‐1; R&D Systems), CXCL1 (Cat# CSB‐E17286m; CUSABIO), MMP‐9 (Cat#CSB‐E08004m; CUSABIO), and TIMP‐1 (Cat#CSB‐E08007m; CUSABIO) were measured using ELISA kits according to the manufacturers’ instructions. In brief, standard samples, cell supernatants, plasma, or BALF supernatants were added to antibody‐coated plates and incubated at 37°C for 120 min. Biotin‐conjugated antibody solution, streptavidin‐HRP solution, and TMB substrate solution were then sequentially added to the wells. After the stop solution was added, the absorbance was measured at 450 nm within 15 min.

### 4.12 Surface Plasmon Resonance (SPR) Assay

SPR experiments were performed according to the manufacturer's protocol. Briefly, equal volumes of EDC (1‐(3‐dimethylaminopropyl)‐3‐ethylcarbodiimide hydrochloride; 0.4 m aqueous solution) and NHS (N‐hydroxysuccinimide, 0.1 m aqueous solution) were mixed and injected at a flow rate of 5 µL/min over 20 min to activate the CM5 sensor chip (Cytiva, 29149603, USA). The EP2 protein was diluted to a final concentration of 50 µg/mL in 10 mm sodium acetate buffer (pH 4.0) and flowed over the activated surface, where it bound to the chip until a response value of 8000 RU was achieved. To block any remaining activated sites, ethanolamine solution was introduced at a flow rate of 10 µL/min for 10 min. The interaction between EP2 and Cer24:1 was examined by injecting 500 nm Cer24:1 in PBST buffer over the EP2‐coupled chip surface. To determine the equilibrium dissociation constant (KD), Cer24:1 was serially diluted to the following concentrations: 0.976, 1.953, 3.906, 7.812, 15.625, 31.25, 62.5, 125, 250, and 500 nm in PBST. The resulting interactions with EP2 were then analyzed using BIA evaluation software.

### 4.13 Western blot (WB) Analysis and Immunoprecipitation (IP)

In the WB experiments, whole‐cell lysates or tissue lysates were prepared using RIPA buffer (Cat# P0013B; Beyotime) supplemented with protease and phosphatase inhibitors (Cat# GRF103; Epizyme). The samples were incubated on ice for 30 min and then centrifuged at 10 000 rpm for 10 min at 4°C. The resulting supernatant was carefully collected, mixed with 5× sample buffer, and boiled for 10 min.

To identify proteins that interact with biotin‐ceramide during IP, CD4^+^ T cells were washed three times with cold PBS and then incubated on ice for 20 min in preprepared IP lysis buffer containing protease inhibitors. The cell lysates were collected by centrifugation at 10 000 × g for 10 min at 4°C, and the supernatant was carefully retained. Equal volumes of the supernatant were incubated with either 10 µm biotin‐ceramide (Cat# S‐300B, Echelon Biosciences) or an equivalent concentration of biotin for 4 h at 4°C with constant rotation. Streptavidin agarose beads (Cat# 17511301; Cytiva) were then added, and the samples were rotated overnight at 4°C. The beads were washed three times with IP lysis buffer, after which 20 µL of 2 × sample buffer was added to the beads. Finally, the samples were boiled for 10 min to prepare them for subsequent analyses.

For WB analysis, the protein lysates were separated via SDS‒PAGE, transferred to PVDF membranes, and incubated overnight at 4°C with the following primary antibodies: anti‐Smpd1 (1:1000, ABclonal, #39147‐1), anti‐Cers2 (1:1000, Abcam, #ab31545), anti‐Sptlc2 (1:1000, Abcam, #ab229330), anti‐Rorγt (1:1000, Proteintech, #29910‐1‐AP), anti‐Jak2 (1:1000, CST, #3230), anti‐p‐Jak2 (1:1000, CST, #8082S), anti‐Stat3 (1:1000, CST, #4904T), anti‐p‐Stat3 (1:1000, CST, #9145T), anti‐EP2 (1:1000, Abcam, #ab167171), anti‐EP4 (1:1000, Proteintech, #24895‐1‐AP), anti‐CXCR4 (1:1000, Abcam, #ab181020), anti‐CREB (1:1000, CST, #9197), anti‐p‐CREB (1:1000, CST, #9198), and anti‐β‐Actin (1:1000, Proteintech, #20536‐1‐AP). The membranes were subsequently incubated with an HRP‐conjugated secondary antibody (goat anti‐rabbit IgG (H+L), 1:5000; CST, #7074P2) and analyzed using a chemiluminescent imaging system. For quantitative analysis, band intensities were measured by densitometry using ImageJ (NIH). For each lane, the signal of the protein of interest was normalized to the corresponding β‐actin signal to correct for minor variations in loading and transfer. Normalized values from at least three independent biological experiments were then averaged and are presented as the mean ± SEM in the bar graphs. The WB images shown in the main figures are representative blots from these independent experiments.

### 4.14 Real‐Time Quantitative PCR (qPCR)

Total RNA was extracted from frozen lung tissue or Th17 cells using the TRIzol reagent (Thermo Fisher Scientific, Waltham, USA). cDNA was synthesized and subsequently analyzed via RT‒qPCR using TB Green Premix Ex Taq II (TaKaRa, Cat# RR820A) on a QuantStudio 5 Real‐Time PCR System (Thermo Fisher Scientific). Gene expression levels were normalized to *Actb*, and relative expression was calculated using the 2^−^^ΔΔCt^ method. The sequences of all primers used for qPCR in this study are provided in Table S3.

### 4.15 Lung Tissue Histopathology

Mouse lung tissues were fixed in 10% formalin, dehydrated, and embedded in paraffin. Sections with a thickness of 5 µm were prepared. These tissue sections were dewaxed, rehydrated, and stained with hematoxylin and eosin (H&E), periodic acid‐Schiff (PAS), and Masson's trichrome. The lung tissue samples were evaluated by two independent, blinded observers.

Airway inflammation in lung tissues was assessed using a modified semiquantitative scoring system adapted from previous studies [48]. For each mouse, multiple nonoverlapping bronchioles (typically 5–8 airways per section) were examined at high power, and peribronchial inflammation scores (0–4) were assigned as follows: 0, normal; 1, few inflammatory cells; 2, a ring of inflammatory cells 1 cell layer deep; 3, a ring of inflammatory cells 2–4 cells deep; and 4, a ring of inflammatory cells > 4 cell layers deep.

Goblet cell hyperplasia was assessed using PAS staining, with grading on the following criteria [49]: 0, no goblet cells; 1, goblet cells < 25% of the epithelial lining; 2, goblet cells 25%‐50% of the epithelial lining; 3, goblet cells 50%–75% of the epithelial lining; and 4, goblet cells > 75% of the epithelial lining. At least five bronchioles per mouse were scored, and the average PAS score was used as a single value for that animal.

Masson's trichrome staining was used to evaluate collagen deposition in the airways, with collagen fibers clearly stained blue. For each mouse, peribronchial regions surrounding medium‐sized airways were imaged at the same magnification. The area occupied by blue‐stained collagen fibers and the total tissue area in each field were quantified using ImageJ/FIJI software (NIH, Bethesda, MD). Collagen deposition was expressed as the percentage of the collagen‐positive area relative to the total peribronchial area and was averaged across multiple fields per mouse to obtain one value per animal.

### 4.16 Immunohistochemistry

Paraffin‐embedded lung tissue sections were dewaxed and rehydrated, followed by treatment with 3% H_2_O_2_ to block endogenous peroxidase activity. To reduce nonspecific antibody binding, the sections were incubated with 5% goat serum for 1 h at room‐temperature. After blocking, the sections were incubated overnight at 4°C with the following primary antibodies: anti‐MPO (1:500, Abcam, #ab208670), anti‐α‐SMA (1:250, Abcam, #Ab5694), and anti‐fibronectin (1:200, Abcam, #ab268020). The following day, the sections were incubated with secondary antibodies at room‐temperature for 30 min.

Images were acquired under a bright‐field microscope at × 400 magnification, using identical exposure settings for all groups. For each mouse, five nonoverlapping images of bronchioles were captured and analyzed using FIJI (ImageJ). For MPO, the percentage of positively stained area relative to the total tissue area in each field was calculated, and the mean value of the five fields was used as the MPO score per animal. For analysis of α‐SMA and fibronectin expression, staining intensity was quantified by calculating the integrated optical density per area (IOD/area) of the positive signal in each field; values from the five fields were averaged to obtain a single IOD/area value per mouse. All image analyses were performed by two investigators who were blinded to group allocation.

### 4.17 Lipidomics Analysis

For targeted lipidomics analysis using UPLC‒MS/MS, aliquots of 100 µL plasma/sputum/exhaled breath condensate/bronchoalveolar lavage fluid or 10 mg tissue samples were homogenized with 400 µL of precooled 80% methanol (HPLC‐grade) containing internal standards (PC 34:0 and Cer 17:0 at 0.2 µg/µL) and 1 mL methyl tert‐butyl ether (MTBE). After vortexing for 10 min, 250 µL ddH_2_O was added followed by 10 min incubation at room temperature. The mixture was centrifuged at 12 000 × g for 10 min (4°C), and the upper lipid phase was transferred to a new 2 mL tube for evaporation under nitrogen stream. The dried extracts were reconstituted in 150 µL methanol (HPLC‐grade) with 10 min vortexing, and the supernatant was collected for analysis (1 µL injection for positive mode; 5 µL for negative mode). Lipid analysis was conducted using an ACQUITY UPLC liquid chromatography system (Waters, Milford, MA, USA) equipped with a UPLC BEH C18 column (1.7 µm, 100 × 2.1 mm ID; Waters). Detection was performed using a 5500 QTRAP mass spectrometer (AB Sciex, Framingham, MA, USA) with a Turbo Ion Spray electrospray ionization source.

Raw data were processed using Analyst 1.6 and quantified with MultiQuant 3.0.2. Lipid species were identified by retention time alignment with internal standards and relatively quantified by peak area ratios. Subsequent statistical analysis was performed using GraphPad Prism (9.0) and MetaboAnalyst 5.0 at http://www.metaboanalyst.ca. We performed orthogonal partial least squares–discriminant analysis (OPLS‐DA) to visualize group separation. VIP scores were calculated from an OPLS‐DA model constructed in MetaboAnalyst 5.0 based on the bias of targeted lipidomic data. For pairwise comparisons of lipid species between groups (e.g., NA vs. healthy controls, or NA vs. non‐NA asthma), *p* values were calculated using the Mann–Whitney U test. Volcano plots were generated in R, and lipids with *p* < 0.05 and fold change ≥ 1.4 or ≤ 0.714 were considered significantly altered.

### 4.18 Transcriptome Sequencing

The experimental and computational workflows for bulk RNA‐seq, including library preparation, quality control, read alignment, quantification, and downstream statistical analyses, are described in detail in the Supporting Methods.

### 4.19 Single‐Cell RNA Sequencing

To examine the expression of GPCRs in T cells, we obtained count matrices from the GSE135779, GSE142637, and GSE162577 datasets in the GEO database and analyzed them using R. Cell clustering on the basis of mRNA expression levels was performed using the Seurat package, and the SingleR package was used to annotate the resulting cell clusters. For the single‐cell sequencing analysis conducted in this study, as previously described [50], mouse lung tissue was processed using collagenase and digestive enzymes. The dissociated cell mixture was filtered through a 70 µm cell strainer, and red blood cells were lysed with RBC lysis buffer. Single‐cell library construction and sequencing were performed by CapitalBio Technology Co., Ltd. (Beijing, China). The full experimental and computational protocols, including detailed library preparation steps, quality control procedures, and parameter settings for each software, are described in the Supporting Methods.

### 4.20 Statistical Analysis

Statistical analyses were performed using GraphPad Prism software (version 9.0) and SPSS software (version 27.0). Data normality was assessed via the Kolmogorov–Smirnov test. For normally distributed data, comparisons between two groups were conducted using the Student's *t* test, whereas one‐way analysis of variance (ANOVA) was employed for comparisons among three or more groups. Continuous variables are presented as the mean ± standard deviation (SD) or mean ± standard error of the mean (SEM) as specified in the corresponding figure legends and/or tables, and Pearson correlation analysis was used to evaluate correlations.

For nonnormally distributed data, nonparametric tests were applied. The Mann–Whitney U test was used for comparisons between two groups, whereas the Kruskal–Wallis test followed by Dunn's post hoc test was used for comparisons among three or more groups. Nonnormally distributed continuous variables are reported as medians with interquartile ranges (IQRs). Spearman correlation analysis was conducted to evaluate associations. A *p*‐value < 0.05 was considered statistically significant. The specific statistical tests, biological replicates, and multiple‐comparisons procedures used for each experiment are described in the corresponding figure legends.

## Author Contributions

C.C., R.J., and W.L.Z. conceptualized the study. H.L., Z.K., Z.M.L., and A.A. developed the methodology. S.Y., Y.Y.G., and L.T.C. conducted the investigation. H.L. and Z.K. performed visualization. C.C. and W.L.Z. acquired funding. C.C., R.J., W.L.Z., and Y.C.S. supervised the work. H.L. wrote the original draft. H.L., C.C., R.J., and W.L.Z. reviewed and edited the manuscript.

## Funding

National Natural Science Foundation of China (82370032 and 82170028 to C.C. and 81902909 to A.A. and 32071178 to R.J.). Beijing Natural Science Foundation (7232205, C.C.). Peking University Third Hospital's Cohort Construction Project (BYSYDL2021020, C.C.). Key Clinical Projects of Peking University Third Hospital (BYSYZD2023009, C.C.). Capital's Funds for Health Improvement and Research (2024‐3‐40917, C.C.). State Key Laboratory Special Fund (2060204, W.L.Z.). Chinese Academy of Medical Sciences Innovation Fund for Medical Sciences (2023‐I2M‐2‐001, 2025 ‐ I2M ‐ KJ ‐ 019, W.L.Z.).

## Conflicts of Interest

The authors declare no conflicts of interest.

## Supporting information




**Supporting File**: advs74819‐sup‐0001‐SuppMat.docx.

## Data Availability

The raw sequencing data reported in this paper have been deposited in the Genome Sequence Archive at the National Genomics Data Center (GSA: CRA023238, CRA023233, and CRA023221; OMIX: OMIX009351 and OMIX010031), which are publicly accessible at https://ngdc.cncb.ac.cn/. The data that support the findings of this study are available upon request from the corresponding author.
