# Supplementary material for: Endogenous Ceramide 24:1 Constrains Th17‐Driven Neutrophilic Inflammation by Antagonizing EP2 Signaling
Source: Adv Sci (Weinh). 2026 Mar 13;13(29):e20695. doi: 10.1002/advs.202520695 (PMC13205653; doi:10.1002/advs.202520695)
Supplement: Supplementary file 1 — Supporting File: advs74819‐sup‐0001‐SuppMat.docx. [file ADVS-13-e20695-s001.docx]

Contents

[Supplementary figures and figure legends 2](#_Toc209185266)

[Supplementary tables 16](#_Toc209185267)

[Supplementary methods 21](#_Toc209185268)

# Supplementary figures and figure legends

**
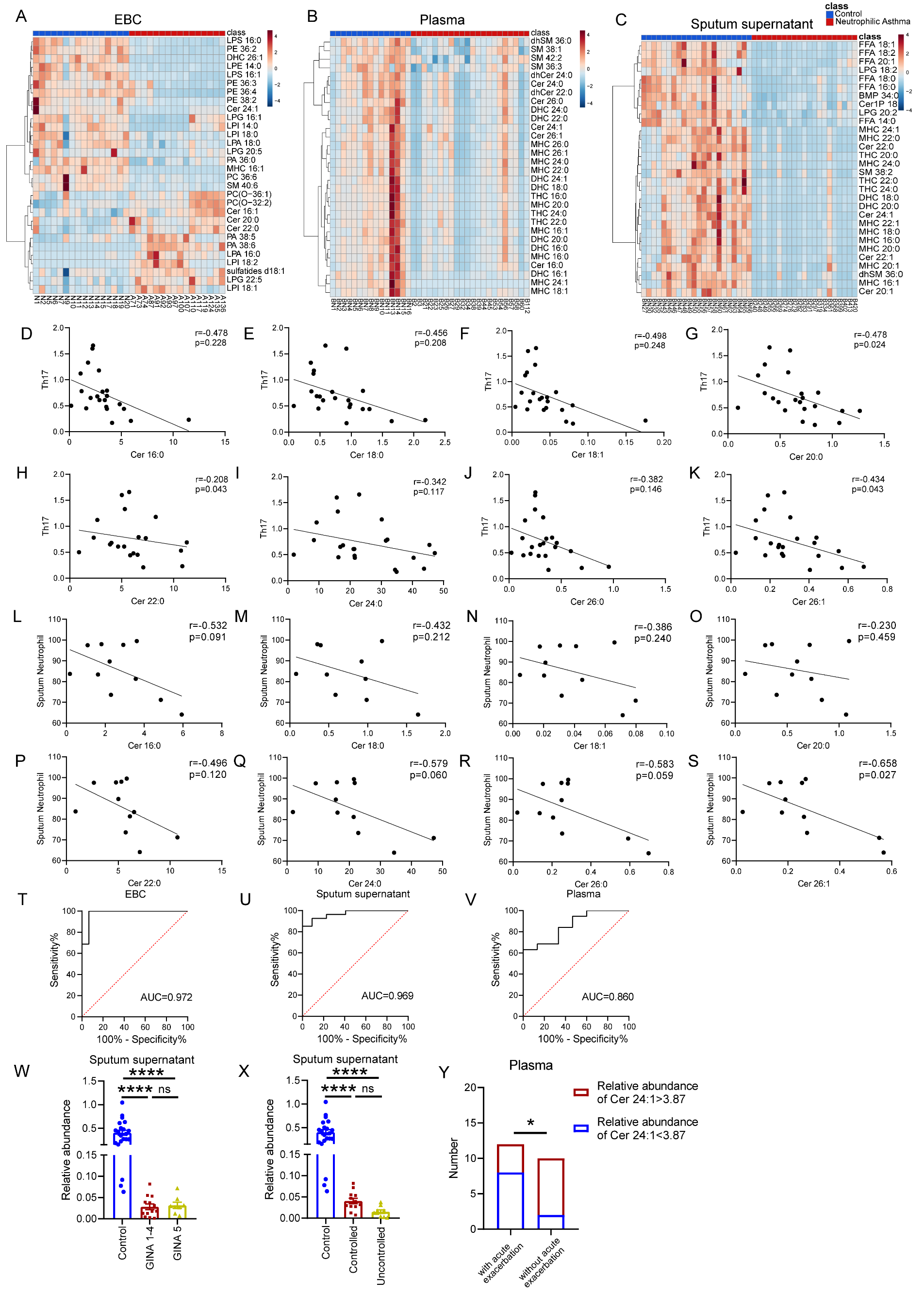
**

Figure S1. The levels of very-long-chain ceramides are negatively correlated with Th17 cell proportions

(A–C) Clustering heatmaps of glycerophospholipids and sphingomyelins in EBC, plasma and induced sputum supernatants from patients with neutrophilic asthma (red) and healthy controls (blue).

(D–K) Correlation analyses between plasma ceramide species and the proportions of Th17 cells in the peripheral blood.

(L‒S) Correlation analyses between plasma ceramide species and the proportions of neutrophils in sputum.

(T–V) ROC curves of Cer24:1 levels in EBC, induced sputum supernatants and plasma from healthy controls and NA patients.

(W, X) Comparison of sputum Cer24:1 levels in neutrophilic asthma patients with well-controlled (ACT > 20, n=12) or uncontrolled (ACT < 20, n=9) symptoms and at GINA stages 1‒4 (n=13) or GINA stage 5 (n=8).

(Y) A lower abundance of Cer24:1 predicted an increased likelihood of acute exacerbations.

The data are presented as the mean ± standard error of the mean (SEM). Statistical analysis was performed *via* the Kruskal‒Wallis test and chi-square test. Spearman correlation analysis was conducted to evaluate associations. **P<*0.05, ** *P<*0.01, *** *P <*0.001, **** *P <*0.0001; ns, not significant.


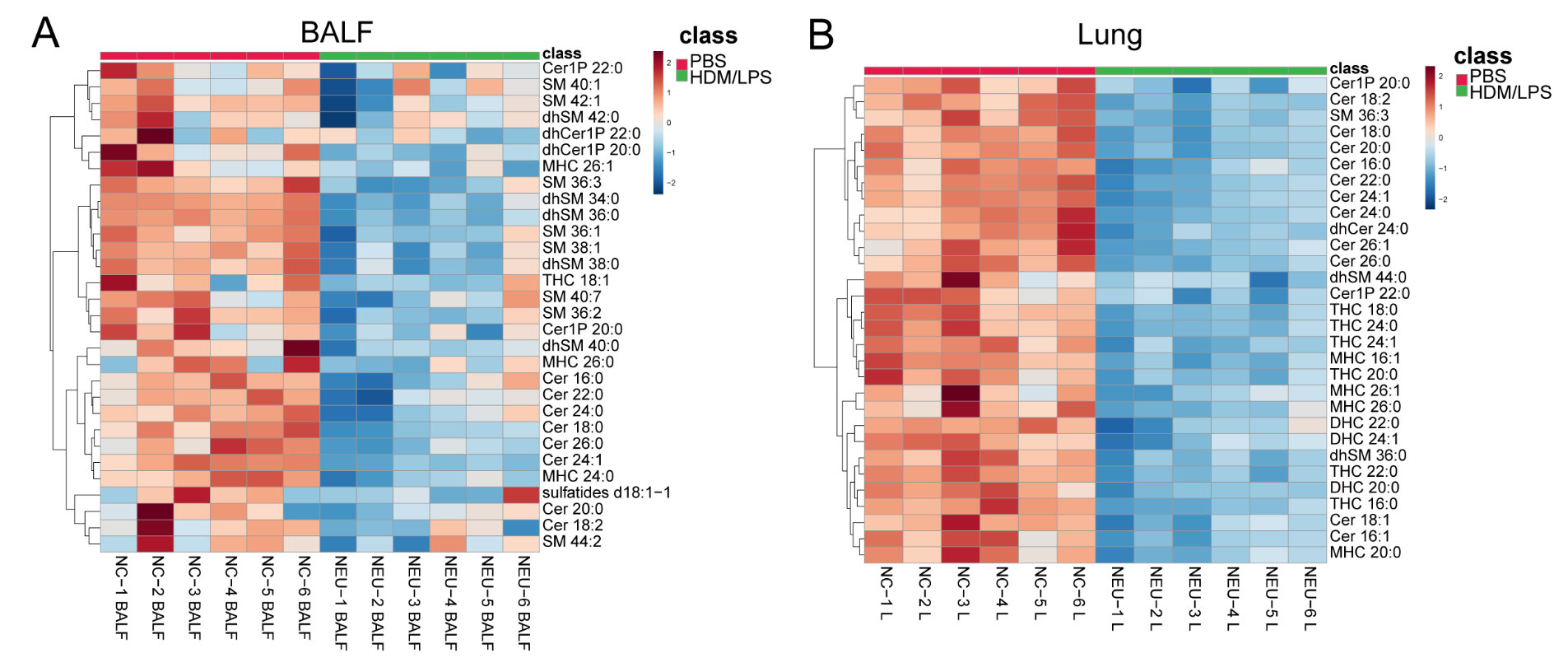


Figure S2. The abundance of very-long-chain ceramides was decreased in HDM/LPS-induced mice.

(A, B) Clustering heatmaps showing differentially abundant sphingomyelins in the BALF, plasma, and lung tissue of PBS-treated mice (red) and HDM/LPS-induced model mice (green).


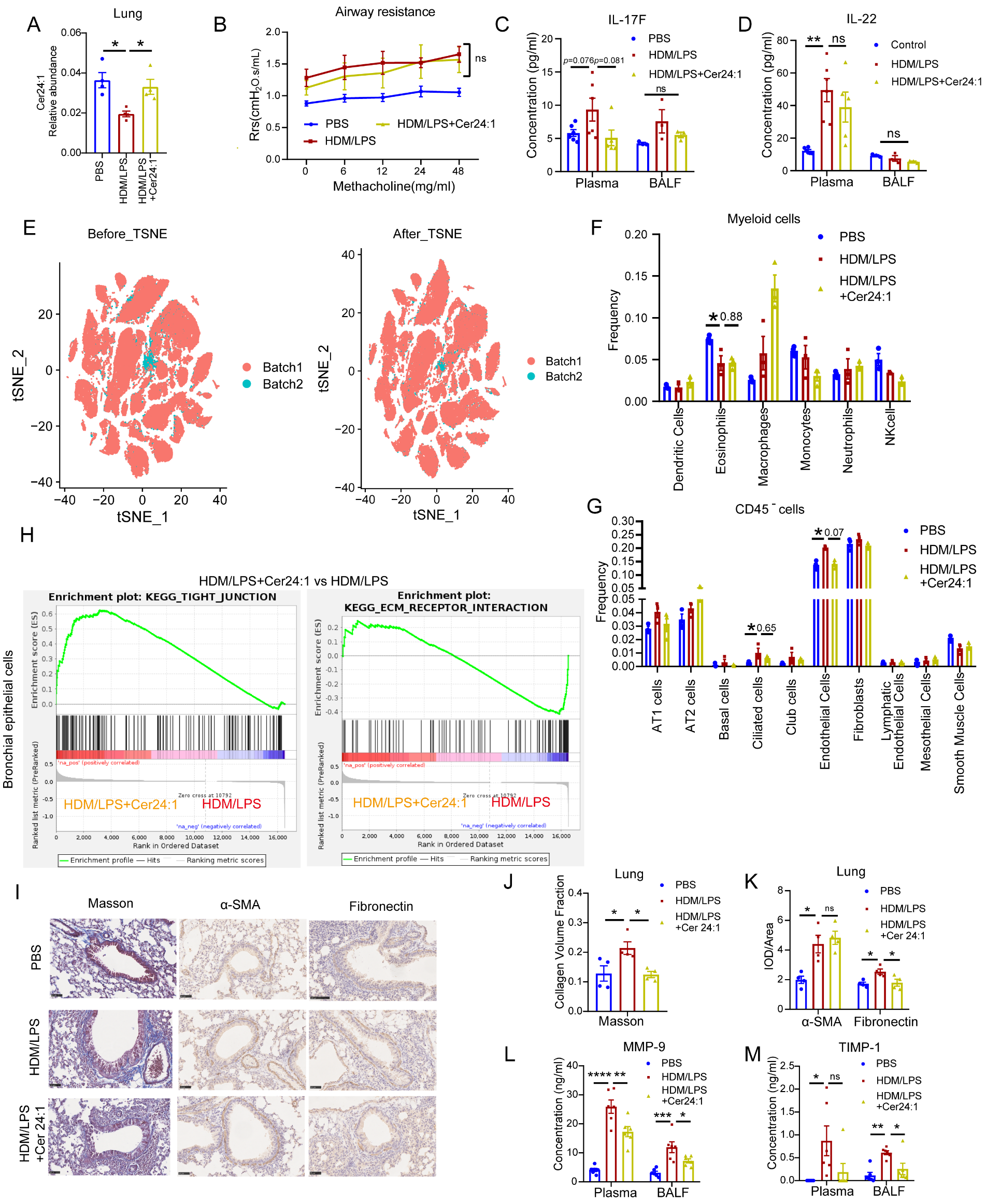


Figure S3. Cer24:1 injection decreased the percentage of Th17 cells in the lungs of the mice.

(A) Relative abundance of Cer24:1 in lung tissue samples from PBS-treated mice (blue), HDM/LPS-induced model mice (red) and Cer24:1-treated model mice (yellow).

(B) Airway resistance in the different groups of mice in response to methacholine. Rrs represents the resistance of the respiratory system.

(C) IL-17F and IL-22 concentrations in plasma and BALF were detected by CBA.

(D) T-SNE plot of single-cell RNA sequencing data from lung tissue.

(E) Single-cell t-SNE plots before and after batch correction. Panels are colored by batch.

(F–G) Statistical analysis of the proportions of different cell types in the lung tissue of the three groups of mice via single-cell RNA sequencing.

(H) GSEA of single-cell RNA sequencing data obtained from bronchial club cells derived from the lung tissues of HDM/LPS-induced and Cer24:1-treated model mice. The analysis revealed that the ECM receptor interaction pathways were inhibited in the Cer24:1-treated mice, whereas the tight junction pathway was activated in the Cer24:1-treated mice.

(I–K) Representative Masson’s trichrome staining and immunohistochemical staining of α-SMA and fibronectin in lung sections, along with a histogram of the pathological score. The scale bar is 50 μm. (n = 4 mice/group).

(L–M) The concentrations of MMP-9 and TIMP-1 in plasma and BALF were detected by ELISA.

The data are presented as the mean ± SEM. For comparisons among three groups, statistical analysis was performed using one-way ANOVA followed by Tukey’s post hoc test (or Kruskal–Wallis test with Dunn’s multiple comparisons test for nonnormally distributed data). **P<*0.05, ** *P<*0.01, *** *P <*0.001, **** *P <*0.0001; ns, not significant.


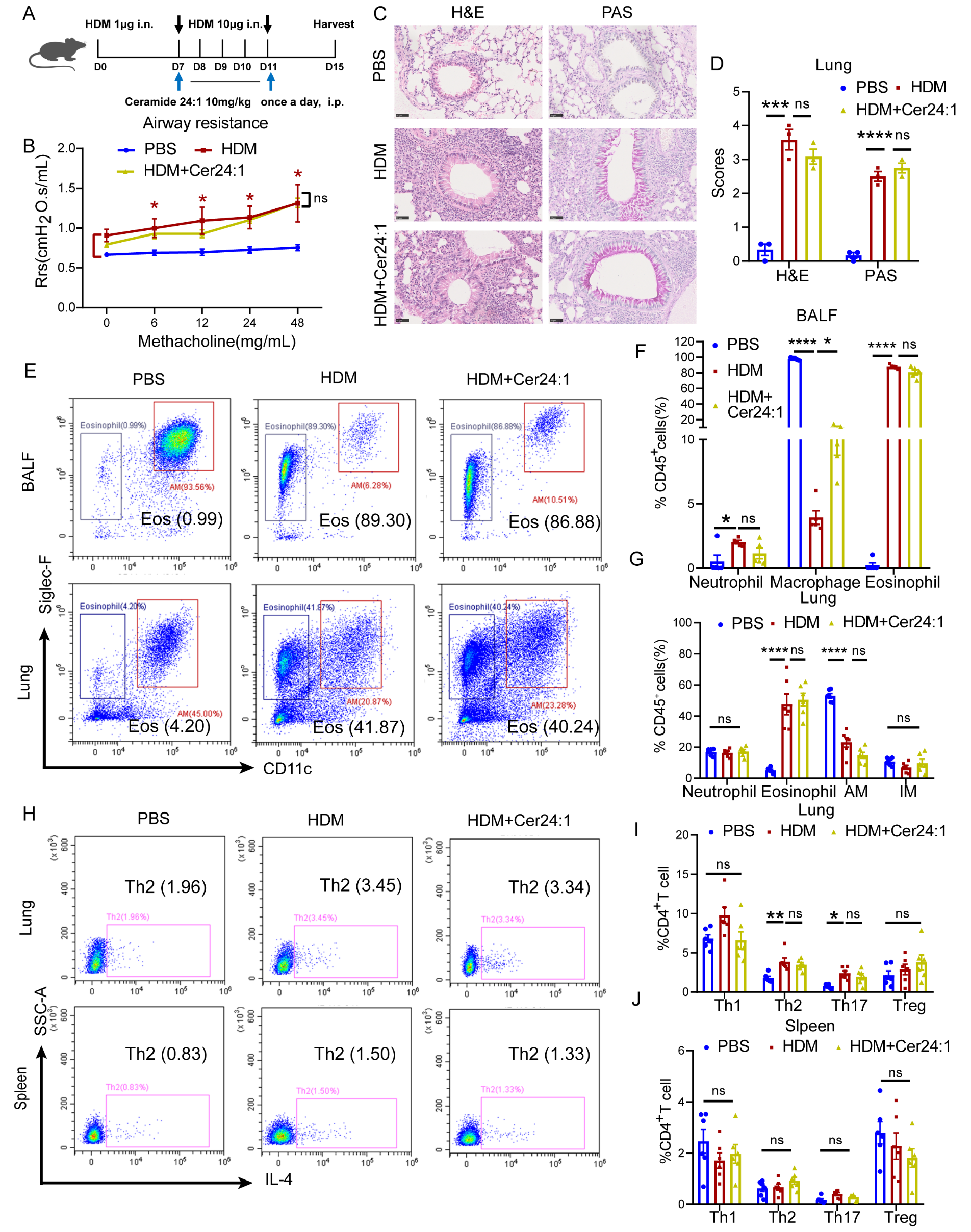


Figure S4. Cer24:1 fails to alleviate eosinophilic asthma

HDM-induced mice were treated with PBS or Cer24:1 for 2 weeks (n = 6 mice/group).

(A) Illustration of the experimental design.

(B) Airway resistance in the different groups of mice in response to methacholine. Rrs represents the resistance of the respiratory system.

(C, D) Representative H&E and PAS images of lung sections, along with a histogram of the pathological score. The scale bar is 50 μm.

(E–G) Flow cytometric analysis and statistical evaluation of neutrophils, eosinophils, alveolar macrophages (AM) and interstitial macrophages (IM) in the BALF (n = 3 mice/group) and lungs (n = 6 mice/group). The proportions of all myeloid cells were analyzed by gating on Zombie Aqua^-^CD45^+^ cells.

(H–J) Flow cytometric analysis and statistical evaluation of Th1, Th2, Th17 and Treg cells in the lungs and spleen (n = 6 mice/group). The proportions of T-cell subgroups were analyzed by gating on Zombie Aqua^-^CD3^+^CD8^-^CD4^+^ T cells.

The data are presented as the mean ± SEM. Statistical analysis was performed using one-way ANOVA followed by Tukey’s post hoc test (or Kruskal–Wallis test with Dunn’s multiple comparisons test for nonnormally distributed data). **P<*0.05, ** *P<*0.01, *** *P <*0.001, **** *P <*0.0001. ns, not significant.


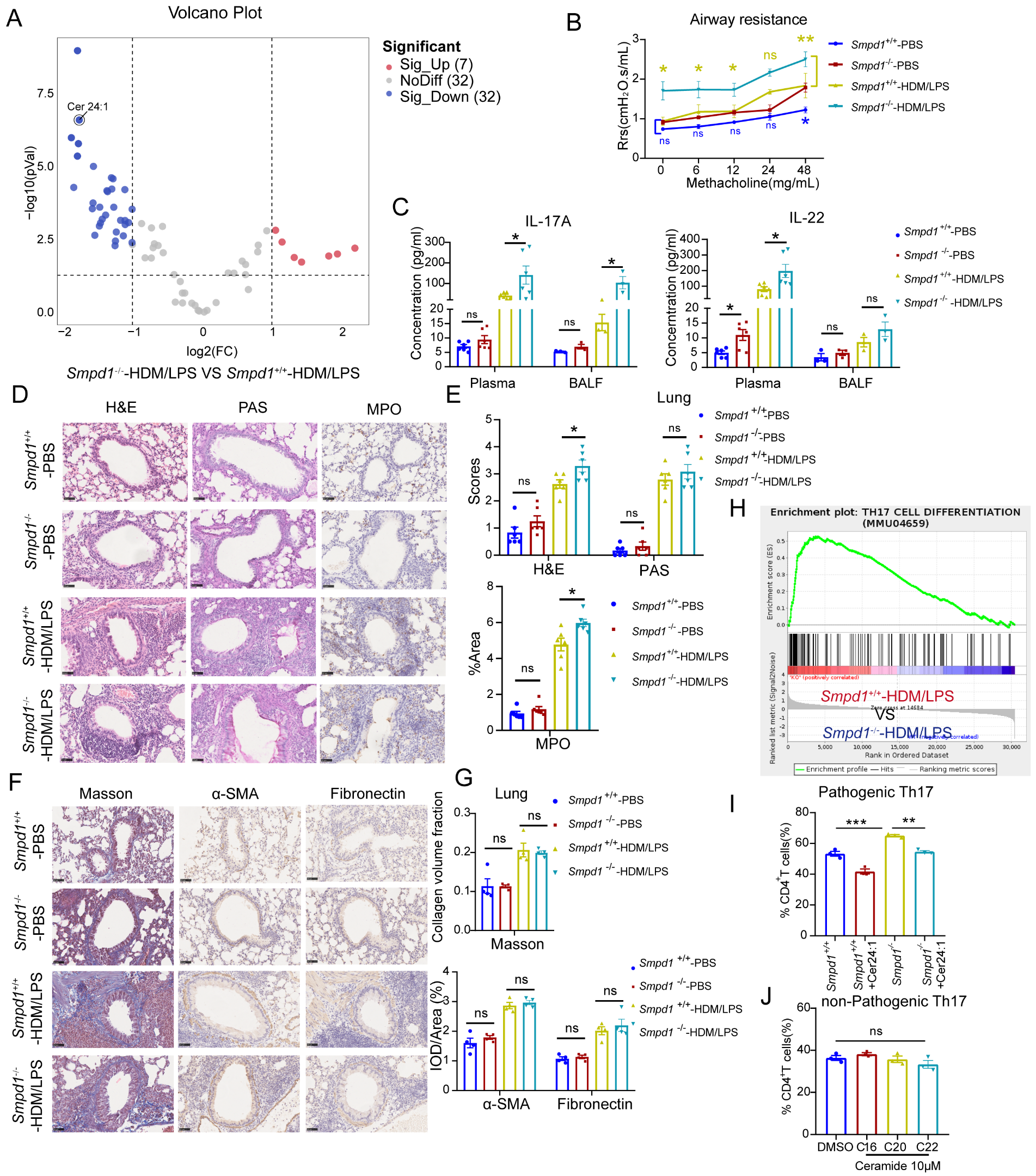


Figure S5. *Smpd1* deficiency accelerated Th17-dominated neutrophilic asthma inflammation in HDM/LPS-induced asthma model mice.

(A) Volcano plot depicting the differentially abundant sphingolipids in the lungs of *Smpd1^-/-^* and *Smpd1^+/+^* HDM/LPS-induced model mice. The colors of the points represent the trends of metabolites between different groups (adjusted *P* values < 0.05 and |log_2_FC| > 1 were considered significantly different between the two groups).

(B) Airway resistance in the different groups of mice in response to methacholine. Rrs represents the resistance of the respiratory system.

(C) IL-17A and IL-22 concentrations in plasma and BALF detected by CBA.

(D–E) Representative H&E, PAS, and immunohistochemical staining of MPO in lung sections, with the histogram showing the pathological scores. Scale bar = 50 μm.

(F–G) Representative Masson’s trichrome staining and immunohistochemical staining of α-SMA and fibronectin in lung sections and a histogram showing the pathological score. The scale bar is 50 μm. (n = 4 mice/group).

(H) GSEA of RNA-seq data obtained from the lung tissues of *Smpd1^-/-^* and *Smpd1^+/+^* HDM/LPS-induced model mice revealed the activation of the Th17 differentiation pathway in *Smpd1^-/-^* HDM/LPS-induced model mice (n = 6 mice/group).

(I) Naïve CD4⁺ T cells isolated from *Smpd1*⁺/⁺ and *Smpd1*⁻/⁻ mice were cultured under pathogenic Th17-polarizing conditions in the presence of vehicle or Cer24:1. The frequency of pathogenic Th17 cells (IL-17A⁺ among CD4⁺ T cells) was quantified by flow cytometry (n = 3).

(J) The percentage of nonpathogenic Th17 cells after C16 (ceramide d18:1/16:0), C20 (ceramide d18:1/20:0) and C22 (ceramide d18:1/22:0) ceramide treatment for 3 days (n = 3).

The data are presented as the mean ± SEM. Statistical analysis was performed using one-way ANOVA followed by Tukey’s post hoc test (or Kruskal–Wallis test with Dunn’s multiple comparisons test for nonnormally distributed data). **P<*0.05, ** *P<*0.01, *** *P <*0.001, **** *P <*0.0001; ns, not significant.


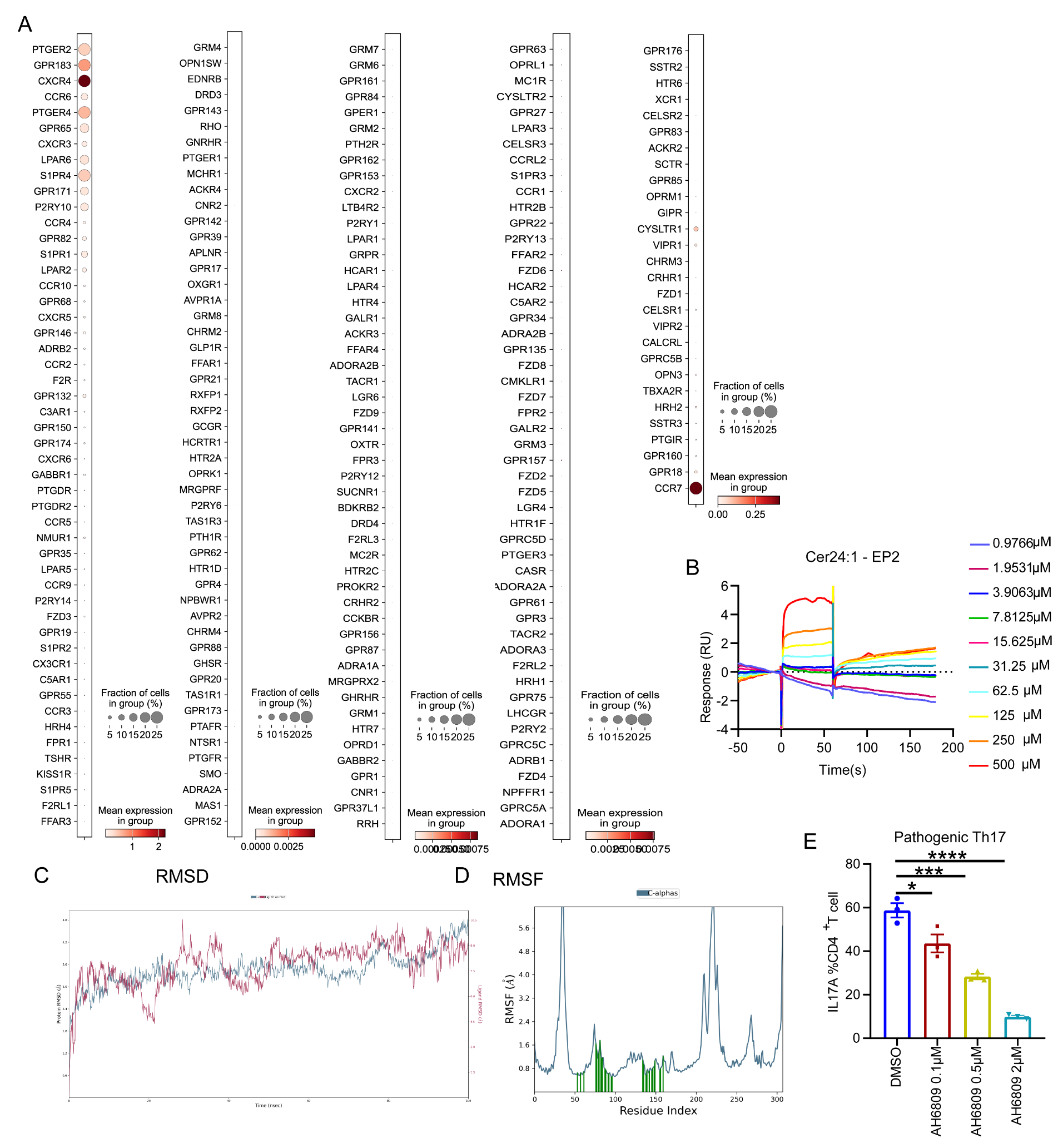


Figure S6. Identification of EP2 as a potential Cer24:1 target through GPCR screening

(A) Single-cell sequencing datasets reveal the expression of G protein-coupled receptor family genes expressed on CD4^+^ T cells.

(B) SPR analysis of the binding between Cer24:1 and EP2.

(C–D) Statistical analysis of the molecular dynamics simulation results using the root mean square deviation (RMSD) and root mean square fluctuation (RMSF).

(E) Flow cytometric analysis and statistical evaluation of the percentage of pathogenic Th17 cells treated with AH6809 (EP2 inhibitor) (n = 3).

The data are presented as the mean ± SEM. Statistical analysis was performed via one-way ANOVA followed by Tukey’s post hoc test. **P<*0.05, ** *P<*0.01, *** *P <*0.001, **** *P <*0.0001; ns, not significant.


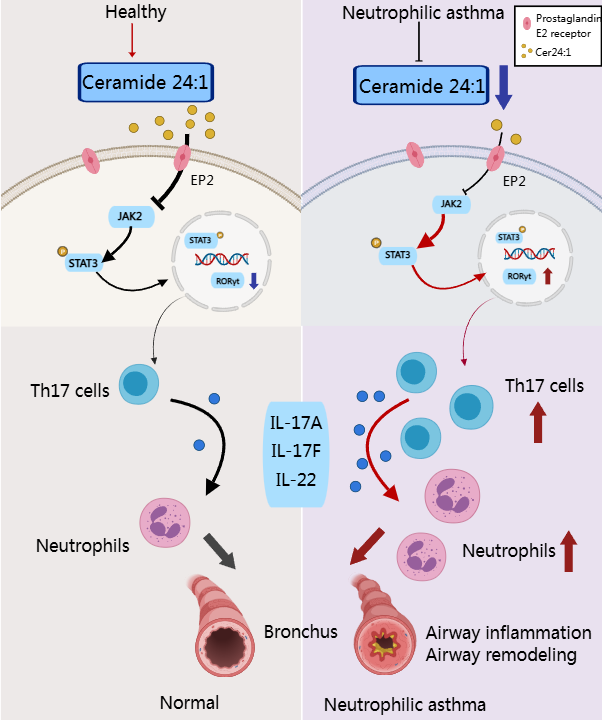


Figure S7. Mechanistic pathways through which Cer24:1 inhibits Th17 cell differentiation by competing with PGE_2_ for EP2 binding in neutrophilic asthma patients.

Schematic illustration of the mechanism through which Cer24:1 competes with prostaglandin E2 (PGE_2_) for binding to the EP2 receptor on CD4⁺ T cells, thereby inhibiting the activation of the JAK2–STAT3–RORγt signaling axis and suppressing Th17 cell differentiation in neutrophilic asthma patients.

This figure was created with MedPeer (medpeer.cn) under an academic license.


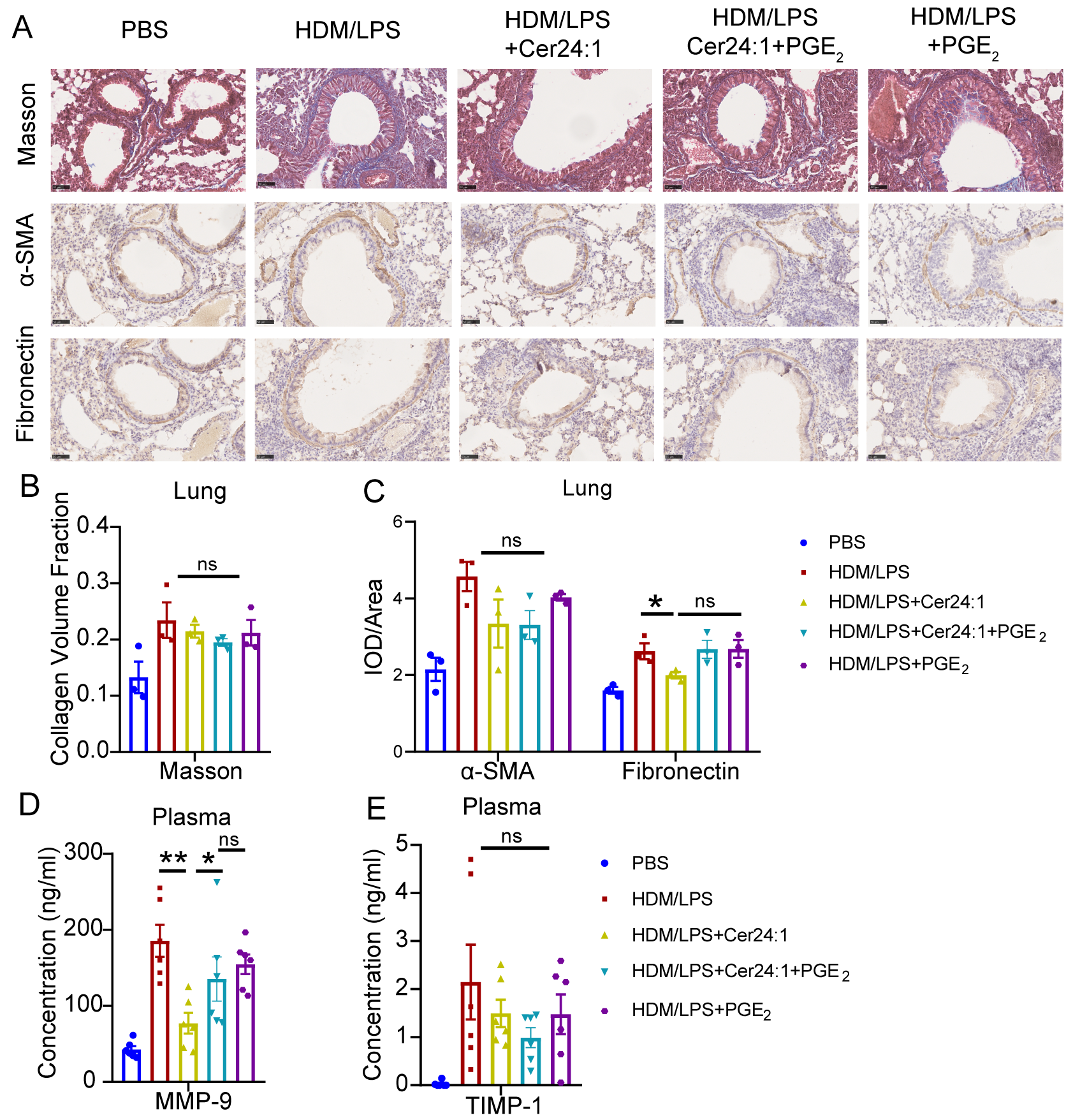


Figure S8. PGE_2_ fails to abrogate the therapeutic effect of Cer24:1 on airway remodeling

(A–C) Representative Masson’s trichrome staining and immunohistochemical staining of α-SMA and fibronectin in lung sections and histograms of the pathological scores. Scale bar = 50 μm (n = 3 mice/group).

(D, E) MMP-9 and TIMP-1 concentrations in plasma and BALF.

The data are presented as the mean ± SEM. Statistical analysis was performed using one-way ANOVA followed by Tukey’s post hoc test (or Kruskal–Wallis test with Dunn’s multiple comparisons test for nonnormally distributed data). **P<*0.05, ** *P<*0.01, *** *P <*0.001, **** *P <*0.0001; ns, not significant.

# Supplementary tables

Table S1. Clinical characteristics of asthma patients and healthy subjects

| Characteristics | Healthy Controls  (n= 53) | Neutrophilic Asthma Patients  (n=85) | Non-Neutrophilic Asthma Patients  (n=60) | *P*^-^value^†^ |
| --- | --- | --- | --- | --- |
| Age (y),  median (IQR) | 47.00 (18.50) | 46.00 (29.00) | 42.00 (24.00) | 0.208 |
| Sex, |  |  |  |  |
| Female (n) | 41 | 55 | 38 | 0.371 |
| Male (n) | 12 | 30 | 22 |  |
| BMI (kg/m^2^), mean (SD) | 23.79 (3.92) | 24.43 (3.29) | 23.65 (3.99) | 0.440 |
| Smoking history (yes), no. (%) | 8 (14.54%) | 15 (17.64%) | 12 (20.00%) | 0.743 |
| Age at onset,  mean (SD) | N/A | 37.31 (18.91) | 30.61 (14.07) | 0.037 |
| Pre-BD FEV1% predicted, mean (SD) | 101.16 (19.51) | 87.42 (15.81) | 89.06 (17.73) | **0.009** |
| Pre-BD FEV1% FVC, mean (SD) | 86.10 (6.02) | 71.90 (9.20) | 73.77 (11.20) | **< 0.001** |
| FeNO (ppb), median (IQR) | N/A | 33.35 (41.25) | 58.00 (33.00) | **0.004** |
| IgE (KU/L) | N/A | 84.34 (140.67) | 77.97 (88.55) | 0.446 |
| Blood eosinophil (×10^9^/L), median (IQR) | 0.11 (0.04) | 0.23 (0.10) | 0.40 (0.25) | **< 0.001** |
| Blood neutrophil (×10^9^/L), median (IQR) | 3.47(1.37) | 3.78 (1.66) | 3.48 (1.70) | 0.121 |
| Sputum total cells, (×10^4^/mL),  median (IQR) | N/A | 49.50 (64.5) | 49.00 (55.30) | 0.968 |
| Sputum macrophage (%), median (IQR) | N/A | 4.7 (12.10) | 20.65 (33.40) | **< 0.001** |
| Sputum lymphocyte (%), median (IQR) | N/A | 0.80 (2.75) | 1.40 (5.72) | 0.066 |
| Sputum neutrophil (%), median (IQR) | N/A | 85.20 (21.75) | 32.75 (24.18) | **< 0.001** |
| Sputum eosinophil (%), median (IQR) | N/A | 1.40 (7.40) | 32.55 (50.33) | **<0.001** |

BMI, body mass index; FEV1, forced expiratory volume in 1 s; FVC, forced vital capacity; FeNO, fractional exhaled nitric oxide; N/A, not available.

*: Continuous variables are presented as the mean (standard deviation, SD) when the data were normally distributed and are presented as the median (interquartile range, IQR) when the data were nonnormally distributed; categorical variables are shown as the no. (%).

†: P values were determined using t tests for normally distributed continuous data or Mann‒Whitney tests for nonnormally distributed continuous data and χ2 tests for categorical data.

Induced sputum was unavailable for 28 asthma participants; thus, sputum-based phenotyping was performed for the remaining 145 asthma participants.

Table S2. Binding energies between Cer24:1 and the top 10 GPCRs.

| Protein_Ligand | Binding_Affinity  (kcal/mol) |
| --- | --- |
| PTGER2_Cer24:1 | -7.064 |
| CXCR3_Cer24:1 | -6.364 |
| GPR171_Cer24:1 | -6.098 |
| PTGER4_Cer24:1 | -5.264 |
| CXCR4_Cer24:1 | -5.018 |
| S1PR4_Cer24:1 | -4.621 |
| GPR65_Cer24:1 | -3.881 |
| LPAR6_Cer24:1 | -3.865 |
| CCR6_Cer24:1 | -3.277 |
| GPR183_Cer24:1 | -3.094 |

Table S3. Primer sequences used in this study.

| Gene | Forward (5'–3') | Reverse (5'–3') |
| --- | --- | --- |
| *Actb* | GTCATCACTATTGGCAACGAGC | TACGGATGTCAACGTCACACTT |
| *Cers1* | GCCACCACACACATCTTTCGG | GGAGCAGGTAAGCGCAGTAG |
| *Cers2* | TACGCGGGATGGAAGAACAC | TGGGCCATACGCAGAATGAA |
| *Cers3* | CCTGGCTGCTATTAGTCTGATG | CTGCTTCCATCCAGCATAGG |
| *Cers4* | TTCTGAACCTCCAGCATGTCG | GATCCTGCACACCCATCCAC |
| *Cers5* | CTGGTCGATGCCTTGGTTCT | CTCGCACCATGTTGTTGACG |
| *Cers6* | CGGCTGGGCATATTTCCTCT | AAAGGGTTCCACTTCCCAGC |
| *Sptlc1* | GAGAGCCTGTCCTTTGGAGTC | AGCAGTATCCTTGACCGGAG |
| *Sptlc2* | AACGGGGAAGTGAGGAACG | CAGCATGGGTGTTTCTTCAAAAG |
| *Sgms1* | GAAGGAAGTGGTTTACTGGTCAC | GACTCGGTACAGTGGGGGT |
| *Sgms2* | TGTCTGTCCTCGGTTGAAGC | GGCCTGACCAATGCTCTCTT |
| *Smpd1* | TGATGGCGGTGAATAGACCTTT | ATGAGAGCTTCCGGGGTAGT |
| *Asah1* | AAAGTCTTCTCACCTGGGTCC | GATTTATGGTGTGCCACGGA |
| *Cerk* | TCGGTACTGGTGTCGGAGAT | CGGTCTTGAAGTCAGGCTCT |
| *Il17a* | GACCCTGATAGATATCCCTCTG | CAGAATTCATGTGGTGGTCCAG |
| *Il17f* | GAGGATAACACTGTGAGAGTTGAC | GAGTTCATGGTGCTGTCTTCC |
| *Rorc* | CCGCTGAGAGGGCTTCAC | TGCAGGAGTAGGCCACATTACA |

# Supplementary methods

Molecular docking

The initial structure of the EP2 protein (UniProt ID: P43116) was prepared using the prepare_receptor4 script in MGLTools 1.4.2, with appropriately assigned Gasteiger partial charges. The three-dimensional structure of ceramide Cer24:1 (CAS: 54164-50-0) was generated using the open-source cheminformatics software package Open Babel 3.0.0, followed by Gasteiger partial charge assignment using the prepare_ligand4 script in MGLTools 1.4.2. Molecular docking was performed with AutoDock Vina v1.2.5, where the docking center was defined as the second-ranked pocket predicted by CavityPlus. The docking box was set as a cubic region with a side length of 25 Å, and 20 conformations were generated as outputs. The optimal conformation, which was chosen on the basis of docking scores and hydrogen bond interactions, was selected for subsequent analysis.

Molecular dynamics simulation

Molecular dynamics simulation was performed using the Desmond 2021.1 Academic Version. The OPLS4 force field was applied, with POPC as the lipid bilayer using the TIP3P water model used. The proteins and small molecules were placed within a cubic water box. The electrostatic and van der Waals interaction cutoff distances were set to 1.0 nm, and a 2-fs time step was used. The simulation was performed at 27°C (300 K) and a pressure of 1.01325 bar. After system configuration, an initial 100 ps Brownian Dynamics NVT simulation was conducted at 10 K, followed by a series of equilibration steps: 12 ps of NVT and NPT equilibration at 10 K, 12 ps of NPT with heavy-atom constraints, and 24 ps of unconstrained NPT. Finally, a comprehensive 100 ns of MD sampling was performed.

Transcriptome Sequencing

Bulk RNA sequencing (RNA-seq) was performed by Beijing Novogene Bioinformatics Technology Co., Ltd. Total RNA integrity and quality were assessed using an Agilent 2100 bioanalyzer (Agilent Technologies, CA, USA), and only high-quality RNA samples were used for library construction. mRNA was isolated from total RNA using oligo-dT magnetic beads, followed by fragmentation, cDNA synthesis, end repair, adaptor ligation, and PCR amplification according to the standard protocol provided by the service provider. Sequencing was performed on an Illumina HiSeq platform.

For data analysis, the raw sequence reads were subjected to quality control and adaptor/low-quality base trimming using fastp. Clean reads were aligned to the mouse reference genome (mm10) to generate gene-level read counts. Gene expression was quantified using featureCounts (v1.5.0-p3), and the resulting count matrix was imported into R (v4.3.1) for downstream analysis.

Differential expression analysis was performed with the DESeq2 (v1.20.0) R package. Raw counts were normalized using the DESeq2 internal normalization procedure, and differentially expressed genes were defined as those with an adjusted P value (Benjamini–Hochberg) < 0.05 and an absolute log₂ fold change (|log₂FC|) ≥ 1.0.

Gene Ontology (GO) and Kyoto Encyclopedia of Genes and Genomes (KEGG) pathway enrichment analyses of differentially expressed genes were carried out using clusterProfiler (v3.8.1). Pathways were considered significant at adjusted P (padj, Benjamini–Hochberg) < 0.05. In addition, gene set enrichment analysis (GSEA) was performed using the GSEA software with species-specific GO and KEGG gene sets. All detected genes were ranked by their differential expression statistics, and gene sets were considered significantly enriched according to the standard criterion FDR (q value) < 0.25.

Single-cell RNA sequencing and data analysis

Single-cell capture and cDNA synthesis were performed using the Single-Cell 3′ Library and Gel Bead Kit v3 (10× Genomics, #1000075) in combination with the Chromium Single-Cell B Chip Kit (10× Genomics, #1000074). scRNA-seq libraries were prepared with the Single-Cell 3′ Kit v3 (10× Genomics) according to the manufacturer’s instructions and sequenced on the Illumina HiSeq X Ten platform. Raw sequencing quality was assessed using FastQC and MultiQC.

The raw FASTQ files were processed with Cell Ranger (v7.2.0; 10× Genomics) to align reads to the mouse reference genome (mm10) and demultiplex barcodes, and generate gene–cell expression matrices. These matrices were imported into Seurat (v3.0) in R for downstream analysis. Low-quality cells with fewer than 200 detected genes or with >25% of the reads mapping to mitochondrial genes were excluded, and genes detected in fewer than three cells were removed.

The remaining high-quality cells were normalized using the Seurat NormalizeData function (log normalization), and highly variable genes (nfeatures = 3000) were identified with FindVariableFeatures. The integrated object was scaled using ScaleData, and principal component analysis (PCA) of the variable genes was performed with RunPCA. To correct for batch effects across samples and conditions, we applied Harmony-based integration by running RunHarmony with sample identity as the batch variable. The Harmony-corrected embeddings were subsequently used for downstream analyses.

For cell clustering and, where indicated, sub-clustering (including monocyte subsets), a shared nearest neighbor graph was constructed using FindNeighbors (dims = 1–30), followed by community detection with FindClusters at a resolution of 0.6. Two-dimensional embeddings were generated with RunTSNE (dims = 1–30) to visualize cell populations.

For refined cell-type annotation, cells were first broadly classified into three major compartments: immune, stromal, and epithelial, based on canonical lineage markers. Each compartment was then subsetted from the Harmony-integrated Seurat object and reprocessed independently. Within each subset, data were re-normalized (NormalizeData), highly variable genes were re-identified (FindVariableFeatures), followed by data scaling (ScaleData) and dimensionality reduction (RunPCA). Neighborhood graph construction (FindNeighbors), clustering (FindClusters), and t-SNE visualization (RunTSNE) were subsequently re-performed to enable higher-resolution sub-clustering, including the identification of immune subpopulations. Cluster-specific marker genes were identified using the FindAllMarkers function, and downstream enrichment analyses were conducted within each subset. Initial cell-type annotation was guided by the automated tool scCATCH and the CellMarker database, and was further refined by manual curation based on established marker genes and published literature.

Differential expression analysis between clusters or conditions was performed using Seurat’s FindMarkers function with the Wilcoxon rank-sum test. Unless otherwise stated, differentially expressed genes (DEGs) were defined by an adjusted *P* value < 0.05 and an absolute log₂ fold change (|log₂FC|) ≥ 0.25. Functional enrichment of DEGs was carried out using clusterProfiler and KOBAS, with the same statistical thresholds (|log₂FC| ≥ 0.25 and padj < 0.05) applied to ensure robustness.

For gene set enrichment analysis (GSEA), we used GSEA software (version 4.2.3) with predefined gene sets from the Molecular Signatures Database (MSigDB, version 7.5). All genes detected in the relevant comparison were ranked according to the difference in mean UMI counts between the target cluster and all remaining clusters. Gene sets were considered significantly enriched according to the FDR criteria recommended by the GSEA software (q value < 0.25).
